# Ion-channel degeneracy and heterogeneities in the emergence of complex spike bursts in CA3 pyramidal neurons

**DOI:** 10.1113/JP283539

**Published:** 2022-10-06

**Authors:** Rituparna Roy, Rishikesh Narayanan

**Affiliations:** Cellular Neurophysiology Laboratory, Molecular Biophysics Unit, Indian Institute of Science, Bangalore, India

**Keywords:** complex spike bursts, computation, degeneracy, dendrite, heterogeneity, hippocampus, ion channels

## Abstract

Complex spike bursting (CSB) is a characteristic electrophysiological signature exhibited by several neuronal subtypes and has been implicated in neural plasticity, learning, perception, anaesthesia and active sensing. Here, we address how pronounced intrinsic and synaptic heterogeneities affect CSB, with hippocampal CA3 pyramidal neurons (CA3PNs), where CSB emergence and heterogeneities are well characterized, as a substrate.We randomly generated 12,000 unique models and found 236 valid models that satisfied 11 characteristic CA3PN measurements. These morphologically and biophysically realistic valid models accounted for gating kinetics and somatodendritic expression profiles of 10 active ion channels. This heterogeneous population of validmodelswas endowedwith broad distributions of underlying parameters showingweak pairwise correlations.We found two functional subclasses of valid models, intrinsically bursting and regular spiking, with significant differences in the expression of calcium and calcium-activated potassium conductances.We triggered CSB in all 236 models through different intrinsic or synaptic protocols and observed considerable heterogeneity in CSB propensity and properties spanning models and protocols. Finally, we used virtual knockout analyses and showed that synergistic interactions between intrinsic and synaptic mechanisms regulated CSB emergence and dynamics. Specifically, although there was a dominance of calcium and calcium-activated potassium channels in the emergence of CSB, individual deletion of none of the several ion channels or *N*-methyl-d-aspartate receptors resulted in the complete elimination of CSB across all models. Together, our analyses critically implicate ion-channel degeneracy in the robust emergence of CSB and other characteristic signatures of CA3PNs, despite pronounced heterogeneities in underlying intrinsic and synaptic properties.

**Figure:**
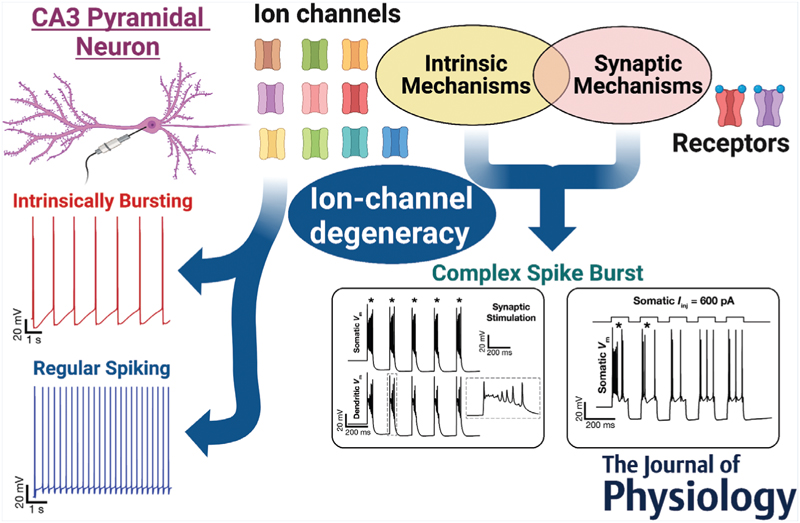
Degeneracy is defined as the ability of disparate structural components to yield the same physiological outcome. With reference to cellular neurophysiology, ion-channel degeneracy is studied as the ability of disparate combinations of ion-channel subtypes to elicit signature cellular-scale functions. Here, we asked whether pyramidal neurons in area CA3 of the hippocampusmanifested ion-channel degeneracy.Our study involved an unbiased stochastic search involving 10 different voltage- and/or calcium-gated ion channels expressed in CA3 pyramidal neurons (CA3PNs). We found a heterogeneous population of 236 morphologically realistic CA3PN models that manifested ion-channel degeneracy in satisfying 11 different signature electrophysiological properties. We classified these models into two subpopulations based on their firing patterns: intrinsically bursting and regular spiking neurons.We triggered complex spike bursting in these neurons either through current injections or through synaptic stimulation involving glutamate receptors. Our analyses unveiled ion-channel degeneracy in the robust emergence of complex spike bursting across different models and protocols.

Degeneracy is defined as the ability of disparate structural components to yield the same physiological outcome. With reference to cellular neurophysiology, ion-channel degeneracy is studied as the ability of disparate combinations of ion-channel subtypes to elicit signature cellular-scale functions. Here, we asked whether pyramidal neurons in area CA3 of the hippocampusmanifested ion-channel degeneracy.Our study involved an unbiased stochastic search involving 10 different voltage- and/or calcium-gated ion channels expressed in CA3 pyramidal neurons (CA3PNs). We found a heterogeneous population of 236 morphologically realistic CA3PN models that manifested ion-channel degeneracy in satisfying 11 different signature electrophysiological properties. We classified these models into two subpopulations based on their firing patterns: intrinsically bursting and regular spiking neurons.We triggered complex spike bursting in these neurons either through current injections or through synaptic stimulation involving glutamate receptors. Our analyses unveiled ion-channel degeneracy in the robust emergence of complex spike bursting across different models and protocols.

## Introduction

Complex spike bursting (CSB) is a signature physiological response that can be elicited by several neuronal subtypes across different brain regions, typically in response to strong depolarizing inputs. Several protocols have been used to trigger CSB, including coincident activation of different synaptic pathways (Bittner et al., 2015; Takahashi & Magee, 2009; Wang et al., 2000; Xu et al., 2012), synchronous synaptic inputs and opposing inhibition (Bastian & Nguyenkim, 2001; Grienberger et al., 2014; Harris et al., 2001; Raus Balind et al., 2019; Royer et al., 2012), coincidence of synaptic inputs and action potentials (Larkum et al., 1999; Magee & Johnston, 1997; Stuart & Hausser, 2001) and somatic and/or dendritic current injections (to elicit intrinsic CSB). A multitude of regulatory mechanisms have been implicated in the emergence of CSB, including dendritic size and arborization (Krichmar et al., 2002; Mainen & Sejnowski, 1996; Narayanan & Chattarji, 2010; van Elburg & van Ooyen, 2010), different combinations of intrinsic properties involved in intrinsic CSB (Cueni et al., 2008; Golding et al., 1999; Hablitz & Johnston, 1981; Hemond et al., 2008; Krahe & Gabbiani, 2004; Lazarewicz et al., 2002; Migliore et al., 1995; Narayanan & Chattarji, 2010; Perez-Reyes, 2003; Raus Balind et al., 2019; Sipila et al., 2006; Su et al., 2001; Swensen & Bean, 2003, 2005; Tazerart et al., 2008; Traub et al., 1991; Vervaeke et al., 2006; Vickstrom et al., 2020; Williams & Stuart, 1999; Wolfart & Roeper, 2002; Wong & Prince, 1978; Xu & Clancy, 2008; Yue & Yaari, 2004), synaptic properties (Bastian & Nguyenkim, 2001; Grienberger et al., 2014; Harris et al., 2001; Raus Balind et al., 2019; Royer et al., 2012) and astrocytic activation (Ashhad & Narayanan, 2016, 2019; Condamine et al., 2018; Kadala et al., 2015; Morquette et al., 2015). Although bursts were traditionally studied primarily with reference to reliable transmission of information, selectivity in transmitted information, and synaptic plasticity (Izhikevich et al., 2003; Krahe & Gabbiani, 2004; Lisman, 1997; Metzen et al., 2016; Sakmann, 2017), CSB and associated dendritic plateau potentials have received renewed attention with recent demonstrations that have implicated them in behavioural time scale plasticity (Bittner et al., 2015, 2017; Magee & Grienberger, 2020; Zhao et al., 2020), perception (Larkum, 2013; Manita et al., 2015; Takahashi et al., 2016, 2020), anaesthesia (Aru et al., 2020; Redinbaugh et al., 2020; Suzuki & Larkum, 2020), active sensing (Lavzin et al., 2012; Ranganathan et al., 2018; Xu et al., 2012) and learning (Doron et al., 2020; Larkum et al., 2022). Despite these broad physiological implications, the question of how neurons generate CSB despite widespread heterogeneities in their synaptic and intrinsic properties has remained unexplored.

Cornu ammonis 3 (CA3) is a subregion of the hippocampus with characteristic recurrent connectivity among pyramidal neurons that manifest signature burst firing (Bilkey & Schwartzkroin, 1990; Hablitz & Johnston, 1981; Hemond et al., 2008; Lazarewicz et al., 2002; Migliore et al., 1995, 1999; Narayanan & Chattarji, 2010; Traub, 1982; Traub et al., 1991; Wong & Prince, 1978; Zeldenrust et al., 2018). The CA3 subregion receives inputs from the dentate gyrus and the entorhinal cortex and has been implicated in spatial navigation, spatialmemory, pattern completion and episodic memory (Andersen et al., 2006; Kesner, 2007; Miles et al., 2014; Nakazawa et al., 2002). The CA3 pyramidal neurons elicit CSB when stimulated with a multitude of stimulus protocols using strong depolarizing inputs to the neuron as a common substrate (Raus Balind et al., 2019).Complex spike bursting is characterized by several consecutive action potentials (APs) within a very small time interval riding on top of a voltage ramp, with AP amplitude reducing within the burst, typically followed by a prolonged period of slow after-hyperpolarization.

In this study, we explore several signature electrophysiological characteristics of CA3 pyramidal neurons, including CSB elicited through multiple protocols, and ask whether this constellation of characteristic electrophysiological measurements constrains the biophysical composition of CA3 pyramidal neurons. A single hand-tuned model would not be sufficient to accommodate the physiologically observed parametric and measurement heterogeneities or to address effectively the question regarding the role of neural heterogeneities in the emergence of CSB. Therefore, we took an unbiased stochastic search approach that has been used effectively in other neural systems (Foster et al., 1993; Goaillard & Marder, 2021; Migliore et al., 2018; Mishra & Narayanan, 2019, 2021a; Mittal & Narayanan, 2018; Prinz et al., 2004; Rathour & Narayanan, 2012, 2014; Rathour et al., 2016; Taylor et al., 2009) to explore the parametric space required for achieving the characteristic properties of CA3 pyramidal neurons effectively. The overall goal here was to understand the biophysical basis of the emergence of CSB in CA3 pyramidal neurons, while carefully accounting for the widespread heterogeneities in the parametric and measurement spaces.

Our extensive search process involving 12,000 morphologically realistic conductance-based models yielded 236 models that manifested signature characteristics of CA3 pyramidal neurons, which were either regular spiking (RS) or intrinsically bursting (IB) neurons. Analyses of these two classes of models unveiled a dominant role for calcium and calcium-activated potassium channels in mediating the distinction between these two classes. We subjected these models to five different protocols for eliciting CSB and studied the different signature measurements from these recordings. Our analyses of model measurements and parameters unveiled the expression of ion-channel degeneracy in CA3 pyramidal neurons in the manifestation of characteristic physiological properties, including CSB.

## Methods

### Modelling a CA3b pyramidal neuron

**Figure 1:**
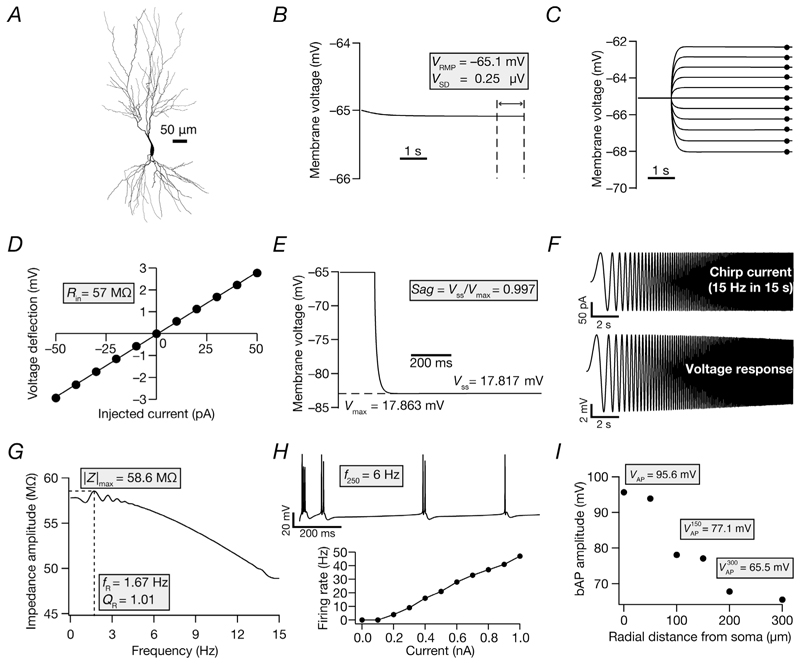
The base model satisfied characteristic intrinsic somatodendritic electrophysiological measurements of CA3 pyramidal neurons *A*, two-dimensional projection of a reconstructed three-dimensional hippocampal pyramidal CA3b neuron model used as the base model. *B–I*, the 11 electrophysiological measurements used to characterize the morphologically realistic model of a CA3b pyramidal neuron, highlighted in grey boxes. *B*, resting membrane potential (*V*_RMP_) was calculated as the mean value of the recorded voltage, in the absence of injected current, within the 5–6 s window. The standard deviation (*V*_SD_)was computed from the same trace spanning the 5–6 s period. All subsequent measurements were performed after an initial delay of 5 s, to allow *V*_RMP_ to reach a steady state. *C*, input resistance (*R*_in_) was calculated from the voltage responses elicited by injecting current pulses of amplitude −50 to +50 pA in steps of 10 pA for 300 ms. *D*, *V–I* plot showing steady-state voltage responses vs. injected current values, obtained from the traces in *C*. The *R*_in_ was computed as the slope of the linear fit to this *V–I* plot. *E*, sag ratio (*Sag*) was measured as the ratio of the steady-state membrane potential deflection (*V*_SS_) to the peak membrane potential deflection (*V*_max_) in the voltage response to a hyperpolarizing current pulse of 250 pA injected for a duration of 800 ms. *F*, impedance-based measurements were obtained from the voltage response (bottom) to a chirp current stimulus (top). *G*, impedance amplitude profile depicting the resonance frequency (*f*_R_) at which the maximal response $\left({\left|Z\right|}_{\text{max}}\right)$ was elicited by the neuron. The resonance strength (*Q*_R_) was calculated as the ratio of the impedance amplitude at *f*_R_ to the impedance amplitude at 0.5 Hz. H, top panel, voltage response to a current pulse of 250 pA injected for 1 s. The firing rate (*f*_250_) was calculated as the number of action potentials (APs) elicited during the period of current injection. Bottom panel, AP firing rate plotted as a function of injected pulse current (1 s duration) amplitude. I, back-propagating action potential (bAP) amplitude plotted as a function of radial distance from the soma. Recordings were obtained at different dendritic locations along the somatodendritic axis after somatic injection of a depolarizing current of 1 nA for 50 ms. The amplitude of the propagating AP at various locations was measured from the respective recordings. Abbreviations: $V_{\text{AP}}^{0}$, AP amplitude at soma; $V_{\text{AP}}^{150}$, bAP amplitude at a dendrite ∼150 μm from the soma; $V_{\text{AP}}^{300}$, bAP amplitude at a dendrite ∼300 μm from the soma.

A morphologically realistic model of a neuron (Fig. 1A) was constructed from the three-dimensional reconstruction (also available as Cell ID 156a in NeuroMorpho.Org) of a CA3b pyramidal neuron (Ascoli et al., 2007; Cannon et al., 1998) with 10 different types of ion channels whose gating kinetics were adopted from previous literature (Migliore et al., 1995). The passive parameters were maintained constant across the entire neuron, with the specific membrane capacitance set at 0.75 μF cm^−2^, specific membrane resistivity (*R*_m_) of 60 kΩ cm^2^ and axial resistivity (*R*_a_) assigned as 200 Ω cm (default values in the base model are provided). The neuronal model was compartmentalized according to the *d_λ_* rule (Carnevale & Hines, 2006) such that iso-potential conditions were maintained within each compartment. The ion channels incorporated into the model were as follows: a fast sodium (NaF) channel; three voltage-gated potassium channels [delayed rectifier (KDR), A-type (KA) and M-type (KM)]; three voltage-gated calcium channels [L-type (CaL), N-type (CaN) and T-type (CaT)]; two calcium-dependent potassium channels [small conductance (SK) and big conductance (BK)]; and a hyperpolarization-activated cyclic nucleotide-gated (HCN) non-specific cation channel.

All ion channels were modelled using Hodgkin–Huxley dynamics (Hodgkin & Huxley, 1952). Sodium and potassium channel currents were modelled using an Ohmic formulation, with reversal potentials set at *E*_Na_ = 50 mV and *E*_K_ = −91 mV. Current through the calcium channels was modelled using the Goldman–Hodgkin–Katz formulation (Goldman, 1943; Hodgkin & Katz, 1949; Migliore et al., 1995). A mobile calcium buffer mechanism and a calcium pump mechanism, both uniformly distributed across the somatodendritic arbor, accounted for calcium decay (Migliore et al., 1995). The total calcium buffer concentration and the total pump density were model parameters that governed calcium dynamics in the model. Longitudinal and radial diffusion (across four annuli) of calcium, the unbound and bound buffers were incorporated into the model definition.

The distribution for all ion channels followed available computational and experimental data from CA3 pyramidal neurons (Kim et al., 2012; Migliore et al., 1995; Narayanan & Chattarji, 2010). The NaF, KDR, CaN, SK, BK and CaT channels were uniformly distributed in the soma and along the dendritic arbor (Lazarewicz et al., 2002; Migliore et al., 1995; Narayanan & Chattarji, 2010). The distribution of CaL channels was perisomatic (≤50 μm from the soma; Migliore et al., 1995; Narayanan & Chattarji, 2010). Different kinetic schemes were used for the proximal (≤100 μm from the soma) and distal distribution (>100 μm) of the KA channel (Migliore et al., 1999; Narayanan & Chattarji, 2010). Our models did not have an explicit axonal compartment (Narayanan & Chattarji, 2010). The M-type potassium channel was distributed perisomatically (≤100 μm), and the kinetics were adopted from earlier studies (Lazarewicz et al., 2002; Migliore et al., 1995). All somatodendritic active and passive parameters were tuned to match the distance-dependent electrophysiological measurements for back-propagating action potential (bAP; Kim et al., 2012) for the CA3b pyramidal neuron. Although there are lines of evidence suggesting the density of HCNchannels in CA3 pyramidal neurons to be lower than that of their CA1 counterparts, the subcellular distribution of HCN channels along the CA3 dendritic arbor is unclear (Raus Balind et al., 2019). Therefore, the general distribution pattern following that of CA1 pyramidal neurons was adopted, with increasing density along the apical dendrite. The HCN channel was distributed in such a way that its density increased as a function of radial distance (*x*) from the soma along the apical dendrite (Lazarewicz et al., 2002) and followed the kinetics as mentioned (Magee, 1998), as follows:

(1) $$g_{\text{h}}(x)={\bar{g}}_{\text{h}}\left[1+\frac{3x}{100}\right]$$

All model parameters and their respective base values are listed and defined in Table 1.

### Intrinsic measurements for model validation

Models were validated against an array of 11 characteristic electrophysiological measurements (sub- and supra-threshold) from CA3 pyramidal neurons (Fig. 1; Table 2). These measurements were computed using well-established procedures (Basak & Narayanan, 2018; Mishra & Narayanan, 2020; Mittal & Narayanan, 2018; Narayanan & Johnston, 2007, 2008; Rathour & Narayanan, 2014; Seenivasan & Narayanan, 2020) and are described below. Resting membrane potential (V_RMP_) was calculated as the mean value of recorded voltage within the 5–6 s window from the 6 s recordings performed in the absence of injected current (Fig. 1B). The standard deviation (V_SD_) was computed from the same trace spanning the 5–6 s period. All sub- and suprathreshold measurements were performed after an initial delay of 5 s, to allow V_RMP_ to reach a steady-state value. Although models stabilized before 1 s, we allowed a 5 s period as a safe range for stabilization to account for extreme parametric combinations thatmight disrupt early stabilization in certain models that were generated by the stochastic search process (see section “Multi-parametric multi-objective stochastic search” below). A long period for stabilization was essential because the neuronal model included detailed calcium-handling mechanisms (pumps and buffers), whichmight contribute to slow evolution of calcium and calcium-activated conductances in extreme scenarios.

Input resistance (*R*_in_) was calculated from the voltage responses elicited by injecting current pulses of amplitude −50 to +50 pA in steps of 10 pA for 300ms (Fig. 1C). The value of Rin was computed as the slope of the linear fit to a *V–I* plot of the steady-state voltage responses vs. injected current values (Fig. 1D). Sag ratio (*Sag*) was measured as the ratioof the steady-statemembrane potentialdeflection (*V*_SS_) to the peak membrane potential deflection (*V*_max_) in the voltage response to a hyperpolarizing current pulse of 250 pA injected for a duration of 800 ms (Fig. 1E).

Impedance-based measurements were obtained from the voltage response to a chirp current stimulus (Fig. 1F). The chirp stimulus used here was a constant-amplitude (100 pA peak-to-peak) sinusoidal current, with frequency increasing linearly from 0 to 15 Hz over a period of 15 s (Basak & Narayanan, 2018; Hemond et al., 2009; Narayanan & Johnston, 2007, 2008). The Fourier transform of the voltage response was divided by the Fourier transform of the chirp current to obtain complex impedance [*Z*(*f*)], as a function of frequency (*f*). Impedance amplitude profile [|Z(f)|] was computed as follows:

(2) $$\left|Z(f)\right|=\sqrt{\text{Re}[Z(f){]}^{2}+\text{Im}[Z(f){]}^{2}}$$

Where $\text{Re}{\left[Z(f)\right]}^{2}$ and $\text{Im}{\left[Z(f)\right]}^{2}$ are the real and imaginary parts of the impedance [*Z*(*f*)], respectively. The frequency at which the value of |*Z*(*f*)| reached its maximum $\left({\left|Z\right|}_{\text{max}}\right)$ was defined as the resonance frequency (*f_R_*; Fig. 1G). The resonance strength (*Q_R_*) was calculated as the ratio of impedance amplitude at *f_R_* to the impedance amplitude at 0.5 Hz.

The firing rate in response to a pulse current injection of 250 pA (*f_250_*) was calculated as the number of APs elicited during the 1 s period of current injection (Fig. 1H). To compute back-propagating action potential (bAP) amplitude as a function of distance from the soma, recordings were obtained at different dendritic locations along the somatodendritic axis after somatic injection of a depolarizing current of 1 nA for 50 ms. The amplitude of the propagating AP at various locations was measured from the first AP in the respective recordings (${\text{V}}_{\text{AP}}^{0}$, AP amplitude at soma; $V_{\text{AP}}^{150}$, bAP amplitude at a dendrite ∼150 μm from the soma; and ${\text{V}}_{\text{AP}}^{300}$, bAP amplitude at a dendrite ∼300 μm from the soma; Fig. 1I).

### Multi-parametric, multi-objective stochastic search

**Figure 2:**
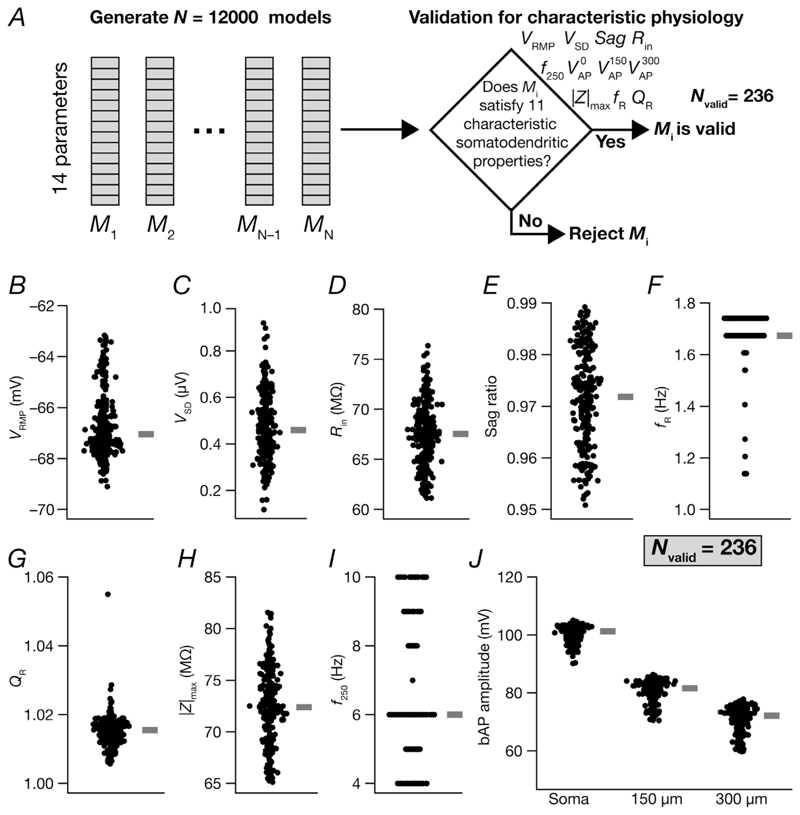
Heterogeneous distribution of signature somatodendritic intrinsic measurements in physiologically valid CA3 pyramidal neuron models obtained from a multi-parametric, multi-objective stochastic search *A*, illustration of the multi-parametric, multi-objective stochastic search (MPMOSS) spanning 14 search parameters (defined in Table 1) and 11 validation measurements (defined in Table 2). *B–J*, beeswarm plots depicting the heterogeneous distribution of the 11 intrinsic somatodendritic measurements from each of the 236 physiologically valid models. All 11 measurements from each of the 236 valid models satisfied all physiological bounds characteristic of CA3 pyramidal neurons (Table 2). Thick lines on the right depict respective median values.

We performed a multi-parametric, multi-objective stochastic search (MPMOSS) on a 14-parameter space that spanned passive properties, ion-channel conductance values and calcium-handling mechanisms (Table 1). The pronounced neuron-to-neuron variability observed in parameters and measurements from biological neurons makes it essential to assess neuronal physiology with a population of heterogeneous models rather than the use of a single hand-tuned model (Marder & Taylor, 2011; Prinz et al., 2003; Rathour & Narayanan, 2019; Sinha & Narayanan, 2022). A variety of approaches have been developed for automated multi-parametric searches towards multi-objective optimization and have been used in physiological assessment of different neuronal subtypes and networks (Bahl et al., 2012; Basak & Narayanan, 2018, 2020; Druckmann et al., 2007; Foster et al., 1993; Friedrich et al., 2014; Goaillard &Marder, 2021; Gouwens et al., 2018; Jain & Narayanan, 2020; Keren et al., 2005; Marder & Taylor, 2011; Menon et al., 2009; Mishra & Narayanan, 2019, 2021b; Mittal & Narayanan, 2018; Navas-Olive et al., 2020; Neymotin et al., 2017; Prinz et al., 2003, 2004; Rathour & Narayanan, 2012, 2014, 2019; Roy & Narayanan, 2021; Seenivasan & Narayanan, 2020; Sinha & Narayanan, 2022; Taylor et al., 2009; Van Geit et al., 2016; Vanier & Bower, 1999). A total of 12,000 unique models were generated randomly by sampling each of these parameters from a uniform distribution that spanned the respective bounds (Table 1). Sub- and supra-threshold physiological measurements of each model were computed, then validated against their respective bounds obtained from electrophysiological recordings from CA3 pyramidal neurons (Table 2). Models with all 11 intrinsic measurements falling within their respective physiological bounds were declared valid (Fig. 2A). The parameters and the measurements of these valid models were then subjected to further analyses exploring heterogeneities and degeneracy.

### Assessing action potential measurements in validated models

**Figure 3:**
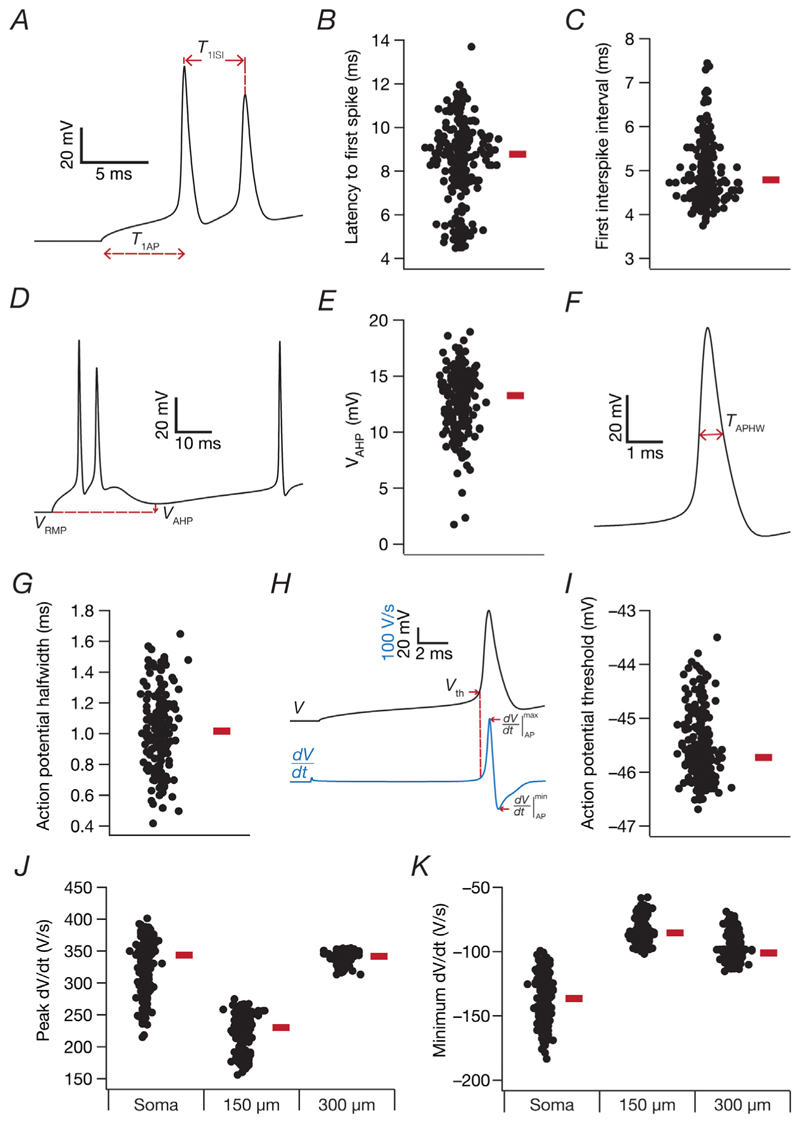
Heterogeneous distribution of action potential measurements in the physiologically valid population of CA3 pyramidal neuron models *A*, illustration of the measurement of latency to first action potential (*T*_1AP_) and first interspike interval (*T*_1ISI_) from voltage traces recorded after the injection of a 250 pA pulse current. *B* and *C*, beeswarm plots depicting the heterogeneous distributions of *T*_1AP_ (*B*) and *T*_1ISI_ (*C*) for all valid models (*N*_valid_ = 236). *D*, illustration of the measurement of spike after-hyperpolarization (*V*_AHP_) from a voltage trace. *E*, beeswarm plot depicting the heterogeneous distribution of VAHP derived for all 236 valid models. *F*, illustration of the measurement of full width at half-maximum of the action potential (*T*_APHW_). The TAPHW was calculated for the 169 regular spiking neurons from the first spike in the model observed after 500 ms of a current injection of 250 pA (for a total period of 1 s). *G*, beeswarm plot for the heterogeneous distribution of TAPHW from 169 regular spiking models. *H*, illustration of the measurement of action potential threshold (*V*_th_) and the maximum $\left(\frac{\text{d}V}{\text{d}t}|\underset{\text{AP}}{\text{max}}\right)$ and the minimum $\left(\frac{\text{d}V}{\text{d}t}|\underset{\text{AP}}{\text{min}}\right)$ values of the action potential derivative (d*V*/d*t*). *I*, beeswarm plot showing the heterogeneous distribution of *V*_th_ for all 236 valid models. *J* and *K*, beeswarm plots for the heterogeneous distribution of the peak and the minimum action potential temporal derivative values for three locations along the somatodendritic arbor (soma and 150 and 300 μm dendritic locations), shown for all 236 valid models. In all beeswarm plots, the thick bar by the side of the plot depicts the respective median.

In models that were validated using the MPMOSS procedure, we computed an additional set of 11 AP measurements (Fig. 3). Various AP-relatedmeasurements (Hemond et al., 2008; Mishra & Narayanan, 2019, 2020, 2021a) were derived from the voltage response of the cell, resting at *V*_RMP_, to a pulse current injection (Fig. 3). As with the other measurements, current injection started after an initial delay of 5 s, during which the membrane potential was allowed to stabilize to a steady-state value. The latency to the first action potential (*T*_1AP_) was measured as the difference between the timing of the first AP since the start of a 250 pA pulse current injection (Fig. 3A). The first interspike interval (*T*_1ISI_)was measured as the temporal difference between the first and second APs immediately after the injection of a 250 pA pulse current (Fig. 3A). Spike after-hyperpolarization (*V*_AHP_) was quantified as the difference between *V*_min_, computed as the minimum voltage attained during the period 0 ≤ *t* ≤ 50 ms for a pulse current of 250 pA injected into the cell, and *V*_RMP_ (Fig. 3D).

Incomplete repolarization associated with bursts hampered accurate quantification of AP width in continuously bursting neurons. Thus, we restricted AP half-width measurements to RS neurons. In addition, given that certain RS neurons manifested bursts immediately after current injection (before switching to RS), we consistently computed the full width at half-maximum of the action potential (*T*_APHW_) from the first spike observed after 500 ms of a current injection of 250 pA (for a total period of 1 s). The *T*_APHW_ wasmeasured as the temporal distance between the two half-maximal points of the AP peak with reference to *V*_RMP_ (Fig. 3*F*). The action potential threshold (*V*_th_) was measured as the voltage value at the time point when the rate of change in voltage with time (d*V*/d*t*) exceeded 20 V s^−1^ (Fig. 3*H*). The temporal derivative of the voltage trace (d*V*/d*t*) was also used to compute the maximum $\left(\frac{\text{d}V}{\text{d}t}|\underset{\text{AP}}{\text{max}}\right)$ and the minimum $\left(\frac{\text{d}V}{\text{d}t}|\underset{\text{AP}}{\text{min}}\right)$ of the AP derivative (Fig. 3H). We computed temporal derivative measurements for three locations along the somatodendritic arbor (soma, 150 μm dendritic location and 300 μm dendritic location; same locations as those for AP amplitude in Fig. 2J).

### Intrinsic bursting and regular spiking models

We delineated intrinsically bursting (IB) neurons from their regular spiking (RS) counterparts by analysing the somatic voltage response to a current pulse of 240 pA injected into the soma for 5.5 s after an initial delay of 5 s (to allow RMP to settle to a steady-state value). Valid neurons derived from the MPMOSS procedure were placed in the IB class if there were three or more APs within 25 ms and if the AP amplitude reduced with successive APs (quantified by the difference between the first and the second APs within the burst). These quantifications were arrived at by visualizing the interspike interval (ISI) histogram of IB *vs*. RS neurons (Frerking et al., 2005; Holt et al., 1996; Selinger et al., 2007).

We performed both linear [principal components analysis (PCA)] and non-linear [*t*-distributed stochastic neighbour embedding (*t*-SNE)] dimensionality reduction techniques on the 12-dimensional active parametric space (Table 1) to understand the distributions of the underlying parameters governing the valid model population. These analyses were performed to assess differences in parametric distributions between the RS and the IB classes of neurons.

### Synaptic model

Colocalized AMPA receptor–NMDA receptor (AMPAR–NMDAR) synapses were placed randomly in the stratum radiatum region along the apical dendrite of the CA3 pyramidal neuron. The number of synapses was set to a default value of 100. The default ratio of NMDARs to AMPARs was set to two, and the ionic currents through these receptors were modelled using the Goldman–Hodgkin–Katz convention (Goldman, 1943; Hodgkin & Katz, 1949; Narayanan & Johnston, 2010). The current through NMDARs reflected their voltage dependence and ionic composition, carried by sodium, potassium and calcium ions, as follows:

(3) $$I_{\text{NMDA}}(v,t)=I_{\text{NMDA}}^{\text{Na}}(v,t)+I_{\text{NMDA}}^{\text{K}}(v,t)+I_{\text{NMDA}}^{\text{Ca}}(v,t)$$

(4) $$\begin{array}{l} I_{\text{NMDA}}^{\text{Na}}(v,t)={\bar{P}}_{\text{NMDA}}\;P_{\text{Na}}s(t)MgB(v)\\ \;\times \frac{vF^{2}}{RT}\left[\frac{{\left[\text{Na}\right]}_{\text{i}}-{\left[\text{Na}\right]}_{\text{o}}\text{exp}(-\frac{vF}{RT})}{1-\text{exp}(-\frac{vF}{RT})}\right] \end{array}$$

(5) $$\begin{array}{l} I_{\text{NMDA}}^{\text{K}}(v,t)={\bar{P}}_{\text{NMDA}}\;P_{\text{K}}s(t)MgB(v)\\ \;\times \frac{vF^{2}}{RT}\left[\frac{{\left[\text{K}\right]}_{\text{i}}-{\left[\text{K}\right]}_{\text{o}}\text{exp}(-\frac{vF}{RT})}{1-\text{exp}(-\frac{vF}{RT})}\right] \end{array}$$

(6) $$\begin{array}{l} I_{\text{NMDA}}^{\text{Ca}}(v,t)={\bar{P}}_{\text{NMDA}}\;P_{\text{Ca}}s(t)MgB(v)\frac{4vF^{2}}{RT}\\ \;\times \left[\frac{{\left[\text{Ca}\right]}_{\text{i}}-{\left[\text{Ca}\right]}_{\text{o}}\text{exp}(-\frac{2vF}{RT})}{1-\text{exp}(-\frac{2vF}{RT})}\right] \end{array}$$

where ${\bar{P}}_{\text{NMDA}}$ defined the maximum permeability of the NMDA receptor. The relative permeability values for Na^+^ and K^+^ were set as *P*_Na_ = *P*_K_ = 1 and for calcium, *P*_Ca_ =10.6. The intra- and extracellular concentrations for the different ions were set as follows: ${\left[{\text{Na}}^{+}\right]}_{\text{i}}=\;18\;\text{mM}$, ${\left[{\text{Na}}^{+}\right]}_{\text{o}}=140\;\text{mM}$, ${\left[{\text{K}}^{+}\right]}_{\text{i}}=140\;\text{mM}$, ${\left[{\text{K}}^{+}\right]}_{\text{o}}=5\;\text{mM}$, ${\left[{\text{Ca}}^{2+}\right]}_{\text{i}}=100\;\text{nM}$ and ${\left[{\text{Ca}}^{2+}\right]}_{\text{o}}=2\;\text{mM}$. These ionic concentrations ensured that the reversal potentials for both AMPA and NMDA were set at 0 mV. The role of magnesium in regulating the activity of the NMDAR was accounted for by the *MgB*(*v*) factor (Jahr & Stevens, 1990), as follows:

(7) $$MgB(v)={\left[1+\frac{{\left[\text{Mg}\right]}_{\text{o}}\;\text{exp}(-0.062v)}{3.57}\right]}^{-1}$$

where ${\left[{\text{Mg}}^{2+}\right]}_{\text{o}}=2\;\text{mM}$. The parameter *s*(*t*) in equations (4–6) governed the kinetics of the NMDA receptor as follows (Jahr & Stevens, 1990):

(8) $$s(t)=a\;\left[\text{exp}\;\left(-\frac{t}{\tau_{\text{d}}}\right)-\text{exp}\;\left(-\frac{t}{\tau_{\text{r}}}\right)\right]$$

where a defined a normalization factor that ensured that 0 ≤ *s*(*t*) ≤ 1. The parameters τ_r_ (= 5 ms) and τ_d_ (= 50 ms) represented the rise time constant and decay time constant, respectively.

The AMPAR current was modelled following the Goldman–Hodgkin–Katz convention and was driven by two ions (sodiumand potassium), as follows:

(9) $$I_{\text{AMPA}}(v,t)=I_{\text{AMPA}}^{\text{Na}}(v,t)+I_{\text{AMPA}}^{\text{K}}(v,t)$$

(10) $$\begin{array}{l} I_{\text{AMPA}}^{\text{Na}}(v,t)={\bar{P}}_{\text{AMPA}}P_{\text{Na}}s(t)\\ \;\times \frac{vF^{2}}{RT}\left[\frac{{\left[\text{Na}\right]}_{\text{i}}-{\left[\text{Na}\right]}_{\text{o}}\text{exp}(-\frac{vF}{RT})}{1-\text{exp}(-\frac{vF}{RT})}\right] \end{array}$$

(11) $$\begin{array}{l} I_{\text{AMPA}}^{\text{K}}(v,t)={\bar{P}}_{\text{AMPA}}P_{\text{K}}s(t)\\ \;\times \frac{vF^{2}}{RT}\left[\frac{{\left[\text{K}\right]}_{\text{i}}-{\left[\text{K}\right]}_{\text{o}}\text{exp}(-\frac{vF}{RT})}{1-\text{exp}(-\frac{vF}{RT})}\right] \end{array}$$

where ${\bar{P}}_{\text{AMPA}}$ defined the maximum permeability of the AMPA receptor, with *P*_Na_ = *P*_K_ = 1. The rise and decay time constants of AMPAR were τ_r_ = 2ms and τ_d_ = 10 ms. When present, these synapses were stimulated by a presynaptic spike train at a frequency of 5 Hz.

### Generation of complex spike bursts

We tried three different classes of protocols to study the emergence of complex spike bursts, adapting the strategies in electrophysiological studies (Raus Balind et al., 2019), as follows:

(1) Large somatic current injections were made. Five pulses of large depolarizing currents with amplitude of 600, 900 or 1200 pA were injected into the cell body for a duration of 100 mswith an interpulse duration of 80ms, after the usual delay period of 5 s for the RMP to attain steady-state values.
(2) Large dendritic current injections were made. Five pulses of large depolarizing currents with an amplitude of 1000 pA were injected into an apical dendritic location (∼150 μm away from the soma) for a duration of 50 ms with an interpulse duration of 50ms, after the usual delay period of 5 s for the RMP to attain steady-state values.
(3) Synaptic stimulation was performed. Synaptic inputs were given by synchronously stimulating 100 colocalized AMPAR–NMDAR synapses with presynaptic spike trains arriving at 5 Hz on dendritic locations randomly distributed over the stratum radiatum.

Complex spike bursts were identified across the five pulses in all the models using characteristic physiological measurements (Table 3): (1) three or more APs in 25 ms within the pulse; (2) maximal reduction in the amplitude of the second AP relative to the first, assessed as the difference between the first AP and the second AP within each pulse; and (3) ramp-like depolarization of the voltage across each pulse, calculated by subjecting the traces to a median filter of a 0.5 s window to remove spikes. The *V*_ramp_ was calculated as the difference of the peak value of this filtered trace from the resting membrane potential (Basak & Narayanan, 2018; Harvey et al., 2009; Roy & Narayanan, 2021). The CSB rate was obtained in the range of zero to one for all the different input configurations, as the ratio between the number of pulses that satisfied each of the CSB criteria and the total number of pulses (Raus Balind et al., 2019). Given that the first pulse always demonstrated a complex spiking burst for all the cases, the CSB rate was computed by taking the remaining four pulses.

### Virtual knockout models

We used the virtual knockout models (VKM) paradigm (Anirudhan & Narayanan, 2015; Basak & Narayanan, 2018; Mishra & Narayanan, 2021b; Mittal & Narayanan, 2018; Mukunda & Narayanan, 2017; Rathour & Narayanan, 2014; Roy & Narayanan, 2021; Seenivasan & Narayanan, 2020) to identify the impact of individual ion channels involved in eliciting distinctive complex spike bursts in CA3 pyramidal neurons. For each of the eight ion-channel subtypes, we set the maximal conductance value to zero across all valid models and assessed the impact of this virtual knockout on different CSB measurements. We used the 900 pA somatic current injection and the synaptic stimulation protocols for inducing CSB and assessed voltage traces for all valid models, independently for each of the eight individual virtual knockouts.We compared these outcomes with the CSB measurements from the respective base models to evaluate the impact of individual ion channels on each CSB measurement.

We assessed the impact of virtual knockout of the NMDAR on CSB measurements obtained with synaptic stimulation across all validmodels by setting theNMDAR maximal permeability to zero during synaptic stimulation (equations 4–6). The AMPAR permeability was intact across models, thus allowing synaptic transmission to occur when the synaptic stimulation protocol for eliciting CSB was used. To understand the role of calcium, mediated through the NMDARs, in triggering CSB in the neurons, we measured local calciumconcentrations across pulses and plotted them in the presence and absence (virtual knockout) of NMDARs.

### Computational details

All simulations were performed using the NEURON programming environment (Carnevale & Hines, 2006) at 34°C. The simulation step size was set as 25 μs. Data analysis and plotting of graphs were done using custom-written codes in MATLAB or IGOR Pro (WaveMetrics, USA). Statistical analysis was performed in R (www.R-project.org). All the data points across all simulations and all models have been reported and represented in the form of beeswarm, scatter or box plots in order to avoid any misleading interpretations arising from merely reporting the summary statistics (Marder & Taylor, 2011; Rathour & Narayanan, 2014).

## Results

We built a biophysically and morphologically realistic base model of a CA3b pyramidal neuron, endowed with characteristic active and passive properties (Table 1) adapted from earlier studies (Migliore et al., 1999; Lazarewicz et al., 2002; Narayanan & Chattarji, 2010) to account for additional constraints on model characteristics (Fig. 1; Table 1). The base model satisfied several signature somatodendritic characteristics of CA3 pyramidal neurons (Fig. 1; Table 2).

### Multi-parametric, multi-objective stochastic search yielded a heterogeneous population of CA3 pyramidal neuron models showing characteristic physiology

The use of a single hand-tuned model for analyses and evaluation introduces biases into the overall conclusions. An alternative is to build a population of models, all of which satisfy the characteristic physiological features of the system under consideration (Marder & Taylor, 2011; Prinz et al., 2003) and assess the phenomena of interest in the population of models (Fig. 2A). Such a population of models, apart from avoiding the obvious biases and disadvantages associated with using a single model for all analyses, also provides a mechanism for assessing heterogeneities and degeneracy in the system under consideration (Anirudhan & Narayanan, 2015; Basak & Narayanan, 2018, 2020; Goaillard & Marder, 2021; Jain & Narayanan, 2020; Mishra & Narayanan, 2019; Mittal & Narayanan, 2018; Mukunda & Narayanan, 2017; Prinz et al., 2004; Rathour & Narayanan, 2014, 2019; Roy & Narayanan, 2021; Seenivasan & Narayanan, 2020; Srikanth & Narayanan, 2015; Shridhar et al., 2022). To generate a population of models, we used a MPMOSS algorithm (Fig. 2A) involving 14 parameters (Table 1) and 11 different objectives (Fig. 1; Table 2). We randomly generated 12,000 unique models and found a small subset of 236 models (∼1.96%) that satisfied all validation criteria involving characteristic features of CA3 pyramidal neurons (Fig. 2B–J; Table 2). These 236 valid models manifested pronounced heterogeneities in their physiological properties, reflective of the heterogeneities observed in CA3 pyramidal neurons (Fig. 2B–J; Table 2).

Our model population was generated by imposing 11 electrophysiological validation criteria (Fig. 2B). How do unvalidated measurements derived from these valid models compare with their electrophysiological counterparts? To assess this, we computed 11 additional electrophysiological measurements derived from action potentials recorded at different parts of the neuronal model and plotted them for the valid model population (Fig. 3). We found these 11 additional measurements to manifest heterogeneities across the population (Fig. 3), with ranges comparable to their physiological counterparts (Hemond et al., 2008; Kim et al., 2012; Raus Balind et al., 2019).

**Figure 4:**
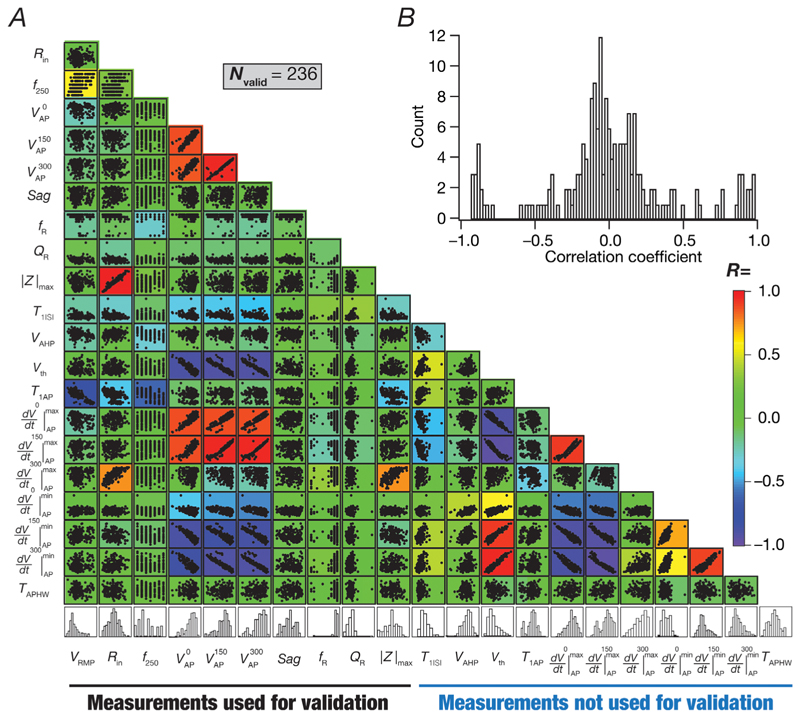
A majority of the pairwise correlations between 21 physiological measurements from the 236 valid models were weak *A*, scatterplot matrix depicting pairwise relationships between 10 intrinsic measurements that were used for validation (VSD was omitted from these analyses) and the 11 action potential measurements that were computed from the valid models. Scatter plots are overlaid on a heat map that shows the correlation coefficient for the respective measurement pair. *N*_valid_ = 236 for all measurements except *T*_APHW_. For *T*_APHW_, *N*_valid_ = 169, because computing the half-maximal width of single action potentials for bursting neurons was confounded by the presence of bursts. The histograms in the last row indicate the span of each measurement across all valid models (for all measurements, *N*_valid_ = 236; for *T*_APHW_, *N*_valid_ = 169). B, histogram of the 210 unique correlation coefficients obtained from the pairwise correlation coefficient matrix shown as a heat map in *A*.

Although we used 11 different measurements for the validation process (Fig. 2) and 11 others for additional assessment (Fig. 3), it was essential to confirm that these measurements were indeed assessing different aspects of CA3 pyramidal neuron physiology. Strong correlations between measurements would imply that the validation process did not involve independent measurements but instead validated the same attribute using disparate measurements. To assess this, we computed Pearson’s correlation coefficient among pairs of all physiological measurements across all valid models (Fig. 4). We found most of these measurements to manifest weak correlations, with only a few pairs showing strong correlations. Among the measurements we used for validation, we found strong correlations between the two subthreshold excitability measures (R_in_ and |Z|^max^) and between amplitudes of APs across the three somatodendritic locations $\left(V_{\text{AP}}^{0},V_{\text{AP}}^{150}\;\text{and}\;V_{\text{AP}}^{300}\right).$ These are expected because the subthreshold excitability measures are dependent on the same parameters and because the bounds on back-propagating APs are with reference to the specific amplitude ranges (of the AP originating at the soma and propagating to different dendritic locations). Among the measurements that were not used for model validation, the expected strong correlations were observed between measures related to the AP temporal derivative (*V*_th_ and the minimum and maximum derivative values at the three different locations) and AP amplitude at the three different locations (Fig. 4).

### Cellular-scale degeneracy in the manifestation of characteristic physiological properties of CA3 pyramidal neuronal models

**Figure 5:**
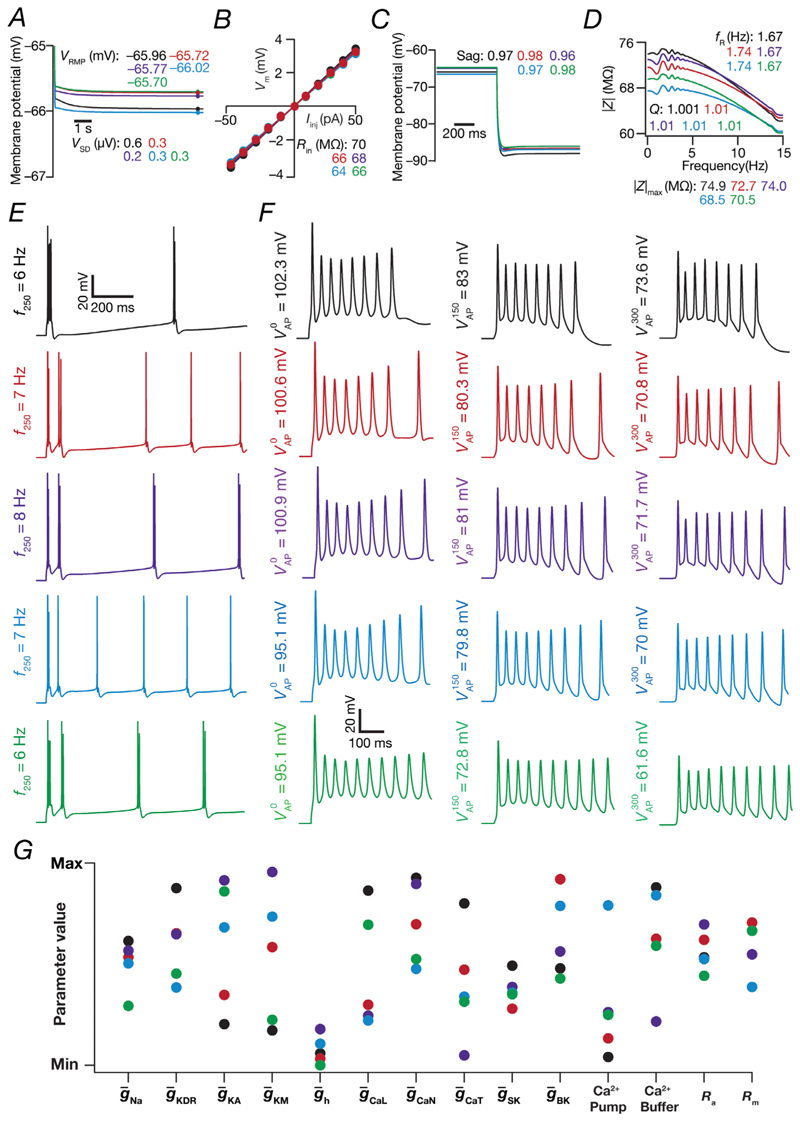
Illustration of degeneracy in the emergence of characteristic physiological measurements in five randomly chosen CA3 pyramidal neuron models *A–F*, voltage traces and 11 associated intrinsic measurements (Table 2) for five distinct valid models chosen from the heterogeneous population of CA3 pyramidal neurons. *G*, plot representing the normalized parameter values (spanning the respective bounds listed in Table 1) for each of these five selected valid models.

Does this CA3 pyramidal neuronal model population require specific parametric values for the emergence of characteristic physiological properties? Could disparate parametric combinations across the parametric space enable the emergence of these signature physiological properties, implying the manifestation of degeneracy? To assess this, we first selected five random models out of 236 whose physiologically relevant intrinsic measurements were very similar to each other (Fig. 5A–F). Although the physiological measurements were very similar and were characteristic of CA3 pyramidal neurons, the underlying parameters manifested widespread heterogeneities spanning their respective minimum-to-maximum ranges (Fig. 5G; Table 1). These observations provided us with a line of evidence that disparate parametric combinations could come together to elicit similar characteristic physiological properties. The ability of disparate parametric combinations to elicit characteristic physiology has been demonstrated with other neuronal subtypes (Goaillard Marder, 2021; Migliore et al., 2018; Mishra & Narayanan, 2019, 2021a; Mittal & Narayanan, 2018; Prinz et al., 2004; Rathour & Narayanan, 2014; Rathour et al., 2016; Taylor et al., 2009). These results show that CA3 pyramidal neurons endowed with signature physiological characteristics could be constructed with disparate parametric combinations.

**Figure 6:**
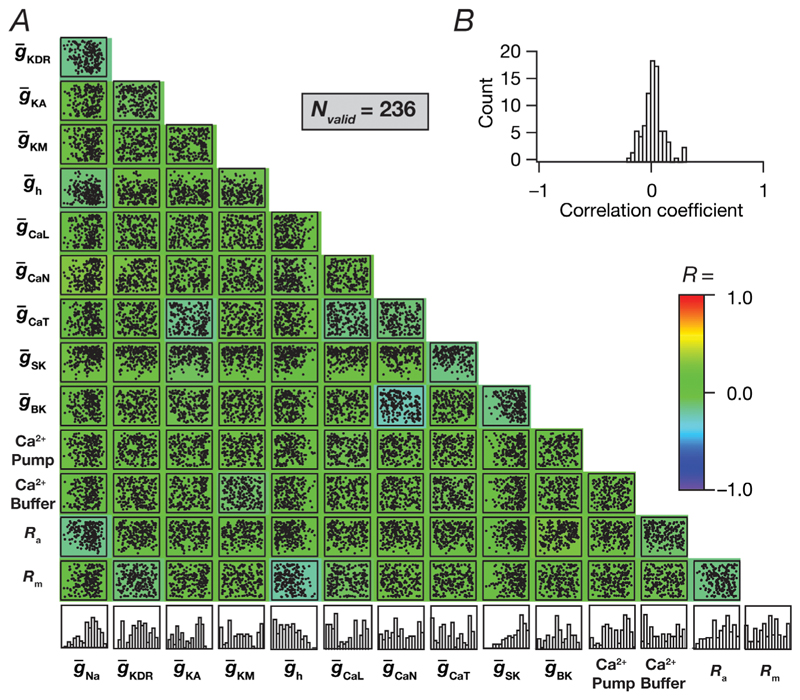
Widespread distributions of individual parameters and weak pairwise correlations between parameters that yielded valid CA3 pyramidal neuron models *A*, scatterplot matrix depicting pairwise relationships between all 14 parameters of the 236 valid models. Scatter plots are overlaid on a heat map that shows the correlation coefficient for the respective pair of parameters. The histograms in the last row indicate the span of each parameter across all valid models. *B*, histogram of the 91 unique correlation coefficients obtained from the pairwise correlation coefficient matrix shown as a heat map in *A*.

These five models provided an illustrative example for the manifestation of cellular scale degeneracy. Were there clusters in the distribution of parameters that governed the 236 valid models? To address this, we plotted the distributions of the 14 parameters that defined these neuronal models and found them to be distributed spanning the range of their respective bounds (Fig. 6A). Moreover, these parameters did not manifest strong pairwise correlations across themselves (Fig. 6), suggesting that changes in one parameter were compensated for by several different parameters and not only one other parameter. It is important to note that the spread of individual parameters spanning their entire minimum-to-maximum range or the lack of strong pairwise correlations among them does not imply that any combination of these parameters could yield valid CA3 pyramidal neuron models. It is crucial to note that only 236 of the 12,000 models were valid, implying that a large proportion of models (∼98.03%) spanning the same parametric range were rejected because they did not satisfy the characteristic physiological properties of CA3 pyramidal neurons. Thus, the lack of structure in the underlying parameters should not be considered as evidence that any parametric combination would yield valid CA3 pyramidal neurons. Instead, the lack of structure in the valid parametric space should be interpreted as evidence for the manifestation of degeneracy, whereby disparate (yet specific) combinations of parameters yield similar physiological properties (Anirudhan & Narayanan, 2015; Basak & Narayanan, 2018, 2020; Das et al., 2017; Edelman & Gally, 2001; Goaillard & Marder, 2021; Jain & Narayanan, 2020; Migliore et al., 2018; Mishra & Narayanan, 2019, 2021a; Mittal & Narayanan, 2018; Prinz et al., 2004; Rathour & Narayanan, 2012, 2014, 2019; Rathour et al., 2016; Roy & Narayanan, 2021; Seenivasan & Narayanan, 2020; Shridhar et al., 2022; Srikanth & Narayanan, 2015; Taylor et al., 2009).

### Heterogeneous CA3 pyramidal neuron population composed of IB and RS models

**Figure 7:**
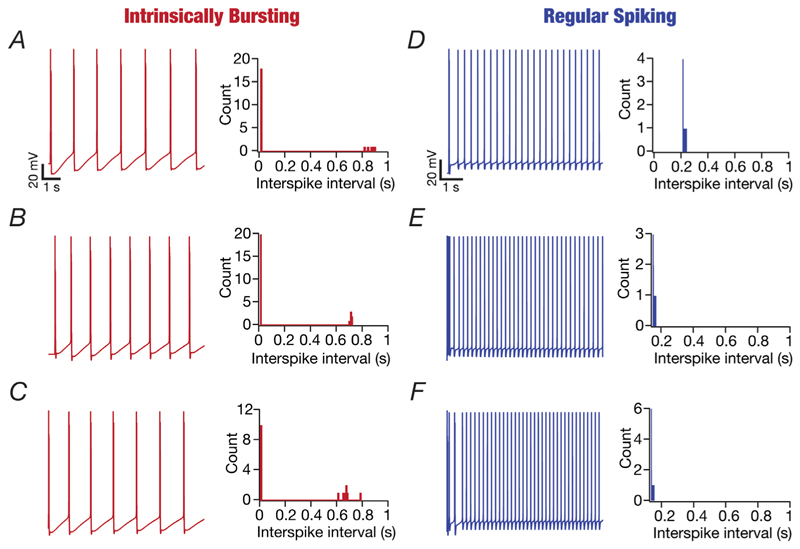
Examples of valid CA3 pyramidal neuron models belonging to the intrinsic bursting and regular spiking classes of firing All voltage traces in this figure were recorded in response to constant current injection of 240 pA for a duration of 5.5 s. The histograms of the interspike intervals were derived from the respective traces. *A–C*, voltage traces from three distinct intrinsically bursting valid models and the associated bimodal interspike interval histograms. *D–F*, voltage traces from three distinct regular spiking valid models and the associated unimodal interspike interval histograms.

The CA3 region contains two classes of pyramidal neurons (Bilkey & Schwartzkroin, 1990; Hablitz & Johnston, 1981; Hemond et al., 2008; Lazarewicz et al., 2002; Migliore et al., 1995, 1999; Narayanan & Chattarji, 2010; Traub, 1982; Traub et al., 1991; Wong & Prince, 1978; Zeldenrust et al., 2018): IB and RS. Intrinsically bursting neurons manifest a burst firing pattern, whereby APs are clustered in distinctly spaced bursts in response to injection with a depolarizing step current. Regular spiking neurons elicit APs with regular ISIs and constant amplitude in response to depolarizing current pulses. We recorded the spiking characteristics of each of the 236 valid models (for a pulse current injection of 240 pA for 5.5 s) and classified them as IB or RS neurons based on their firing profiles and histograms of their ISIs (Fig. 7). Of the 236 valid models, 67 models (∼28.38%) were classified as IB neurons (e.g. Fig. 7A–C; bimodal ISI histograms), and the remaining 169 models (∼71.62%) showed regular spiking behaviour (e.g. Fig. 7D–F; unimodal ISI histograms).

**Figure 8:**
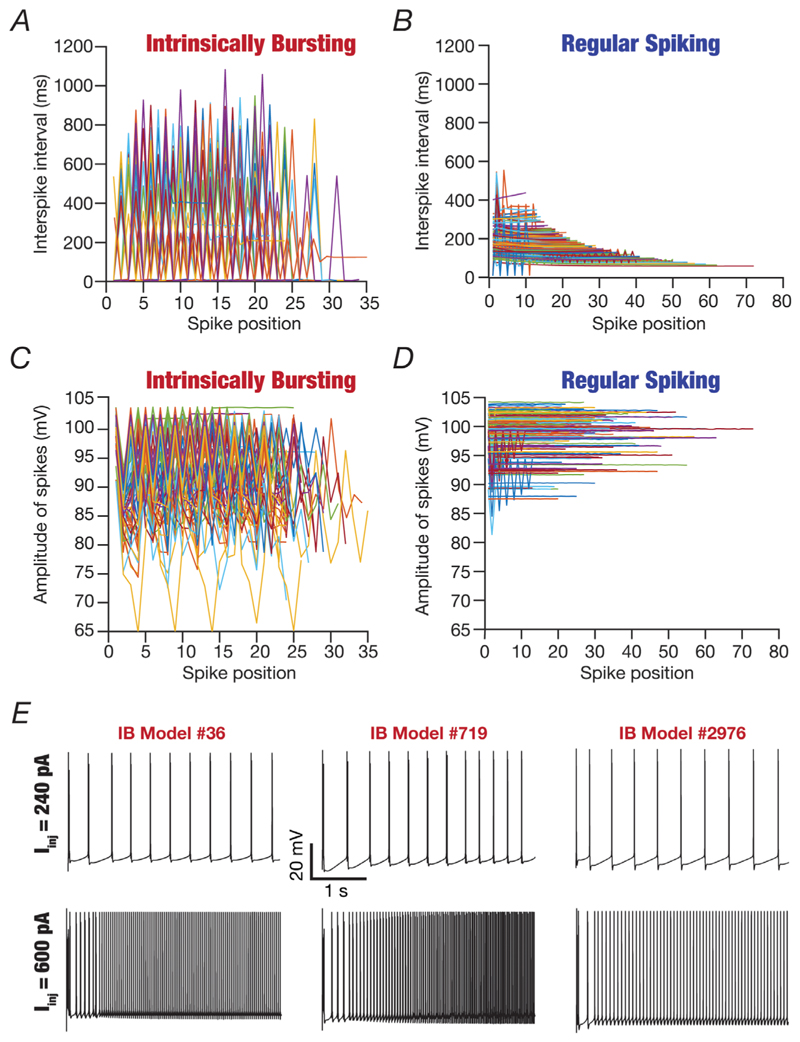
Temporal evolution profiles of spike amplitude and interspike interval delineated the intrinsically bursting and the regular spiking populations *A* and *B*, plots showing the temporal evolution of interspike interval for all intrinsically bursting (*A*) and regular spiking (*B*) neurons in response to the 240 pA current injection for a period of 5500 ms, plotted as a function of spike position within the voltage response. *C* and *D*, same as *A* and *B*, but with plots depicting the temporal evolution of action potential amplitude (difference between peak action potential amplitude and resting membrane potential). *E*, illustrative examples of three intrinsically bursting (IB) neurons transitioning to the regular spiking mode of firing with an increase in the amplitude of injected current. The traces in the top row manifest bursting, and those in the bottom row show regular single spikes for the same model.

The temporal evolution profiles of ISIs as a function of spike position manifested characteristic switches between high and low values for IB neurons (Fig. 8A) but were characteristically constant for RS neurons (Fig. 8B). A small subset of RS neurons showed spike frequency adaptation at the assessed current injection value (240 pA). A characteristic electrophysiological feature of CA3 pyramidal neurons manifesting intrinsic bursting is the continual reduction in AP amplitude within a burst (Bilkey & Schwartzkroin, 1990; Hablitz & Johnston, 1981; Hemond et al., 2008; Lazarewicz et al., 2002; Migliore et al., 1995, 1999; Narayanan & Chattarji, 2010; Traub, 1982; Traub et al., 1991; Wong & Prince, 1978; Zeldenrust et al., 2018). Consistent with this, we observed that IB neurons showed a reduction in amplitude by ∼10 mV (maximum ∼35 mV in one model) across models within the burst and recovery back to a large-amplitude AP at the beginning of the next burst (Fig. 8C). In contrast, the AP amplitude remained constant for most RS neurons, although some RS neurons showed burst-like characteristics during the initial part of the temporal evolution (Fig. 8D). Despite the manifestation of these characteristic features associated with amplitude and ISI profiles, neurons within the IB and the RS subclasses manifested pronounced neuron-to-neuron variability (Fig. 8), further emphasizing the need to use populations of models in assessing neuronal physiology. Furthermore, consistent with prior observations on CA3 IB pyramidal neurons (Migliore et al., 1995; Narayanan & Chattarji, 2010; Su et al., 2001; Yi et al., 2017), we noted that an increase in injected current amplitude in IB neurons resulted in their switch to regular spike firing mode (Fig. 8E).

**Figure 9:**
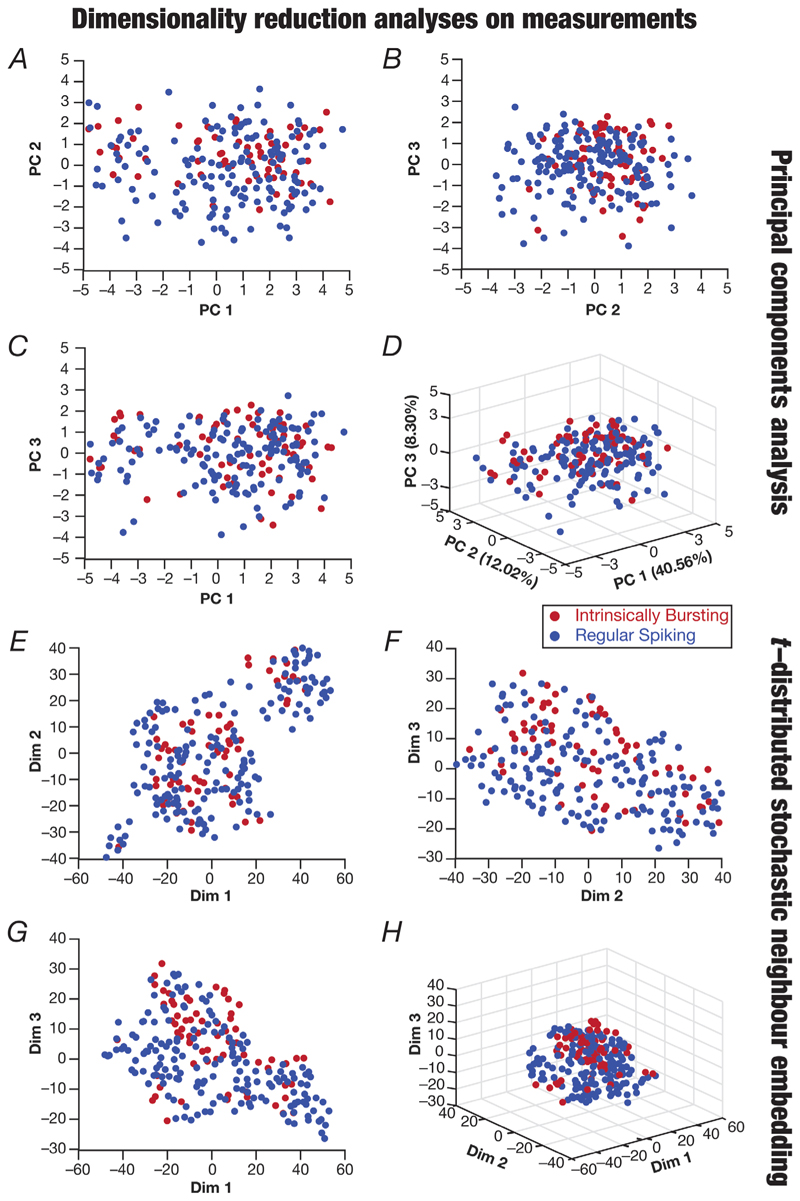
Dimensionality reduction of the electrophysiological measurement space for regular spiking and intrinsically bursting models unveiled overlapping distributions *A*–*D*, principal components analysis performed on the 20 electrophysiological measurements (same set of measurements as in Fig. 4, but action potential width was excluded from these analyses because values for TAPHW were calculated only for the regular spiking neurons) of the 236 valid models. Shown are pairwise plots between the first three principal dimensions (*A*–*C*) and a three-dimensional plot showing all three principal components (*D*). The percentage variance explained by each dimension is provided in *D* along each axis. *E*–*H*, dimensionality reduction results from t-distributed stochastic neighbour embedding (t-SNE) performed on 20 measurements, showing pairwise (*E*–*G*) and three-dimensional (*H*) plots.

**Figure 10:**
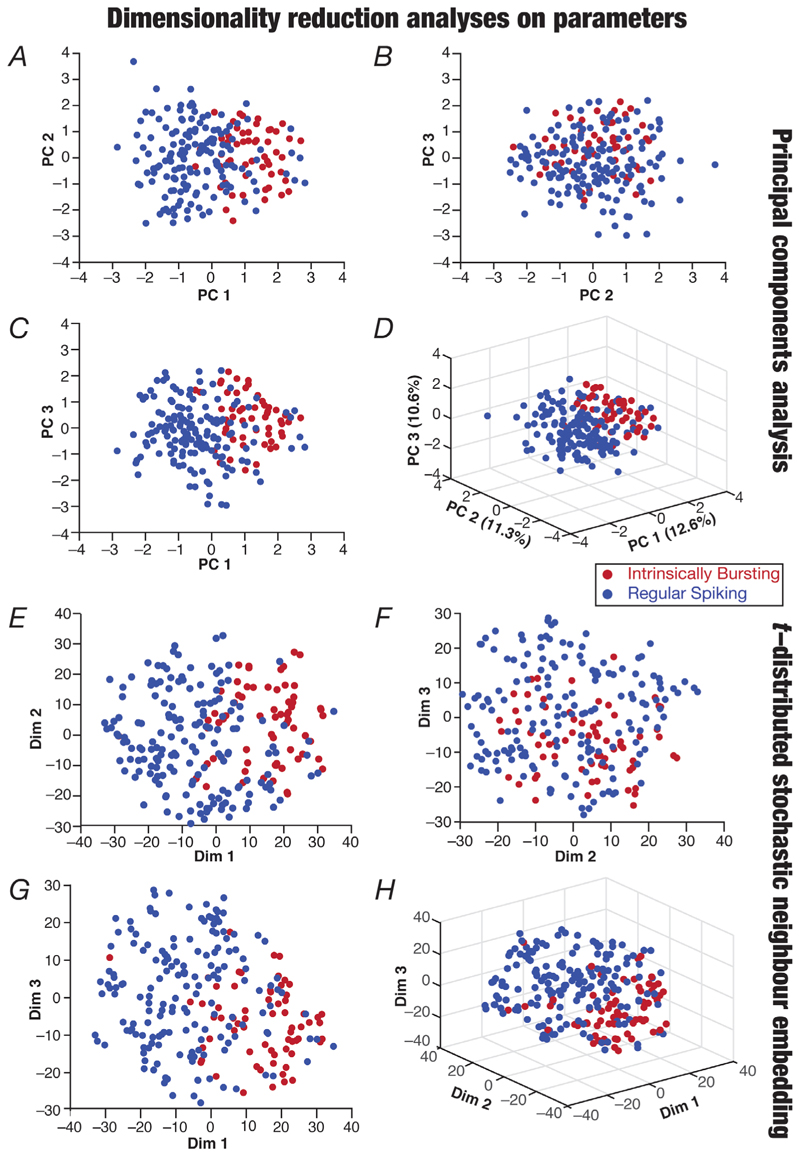
Localization of regular spiking and intrinsically bursting models within a reduced-dimensional active parametric space *A*–*D*, principal components analysis performed on the 12 active parameters of the 236 valid models. Shown are pairwise plots between the first three principal dimensions (*A*–*C*) and a three-dimensional plot showing all three principal components (*D*). The percentage variance explained by each dimension is provided in *D* along each axis. *E*–*H*, dimensionality reduction results from t-distributed stochastic neighbour embedding (t-SNE) performed on 12 parameters, showing pairwise (*E*–*G*) and three-dimensional (*H*) plots.

The 22-dimensional measurement space that was used earlier to validate (Fig. 2) and assess (Fig. 3) the overall population of CA3 pyramidal neurons did not include the ISI distribution (Fig. 7) or the temporal evolution profiles of spike characteristics (Fig. 8). Our classification of IB *vs*. RS neurons was based on additional measurements involving the bimodality of the ISI histogram (Fig. 7), with the ISI and amplitude temporal profiles showing characteristic distinctions that delineated these two classes of neurons (Fig. 8). Did IB and RS neurons occupy distinct subspaces within the measurement space (in Fig. 4), which was generic enough to yield two distinct subclasses of CA3 pyramidal neurons based on additional sets of measurements? To address this, we used either PCA or *t*-SNE and visualized the two classes of neurons in the reduced dimensional measurement space (Fig. 9). We found overlapping distributions of IB and RS subpopulations, with no obvious clustering observed in the measurement space (Fig. 9) that did not include measurements (Figs 7 and 8) that were able to discriminate clearly between these classes. We also performed dimensionality reduction analyses on the active parameters of the 236 models using PCA or *t*-SNE and visualized the identified RS and IB neurons on the reduced dimensional space (Fig. 7). Although no distinct non-overlapping clustering was observed, we found the IB and RS neurons to reside predominantly in different parts of the reduced dimensional space with both dimensionality reduction techniques, suggesting important differences in the parametric space associated with the two classes of neurons (Fig. 10).

### Calcium and calcium-activated potassium channels were the dominant contributors to the distinction between IB and RS neuronal classes

**Figure 11:**
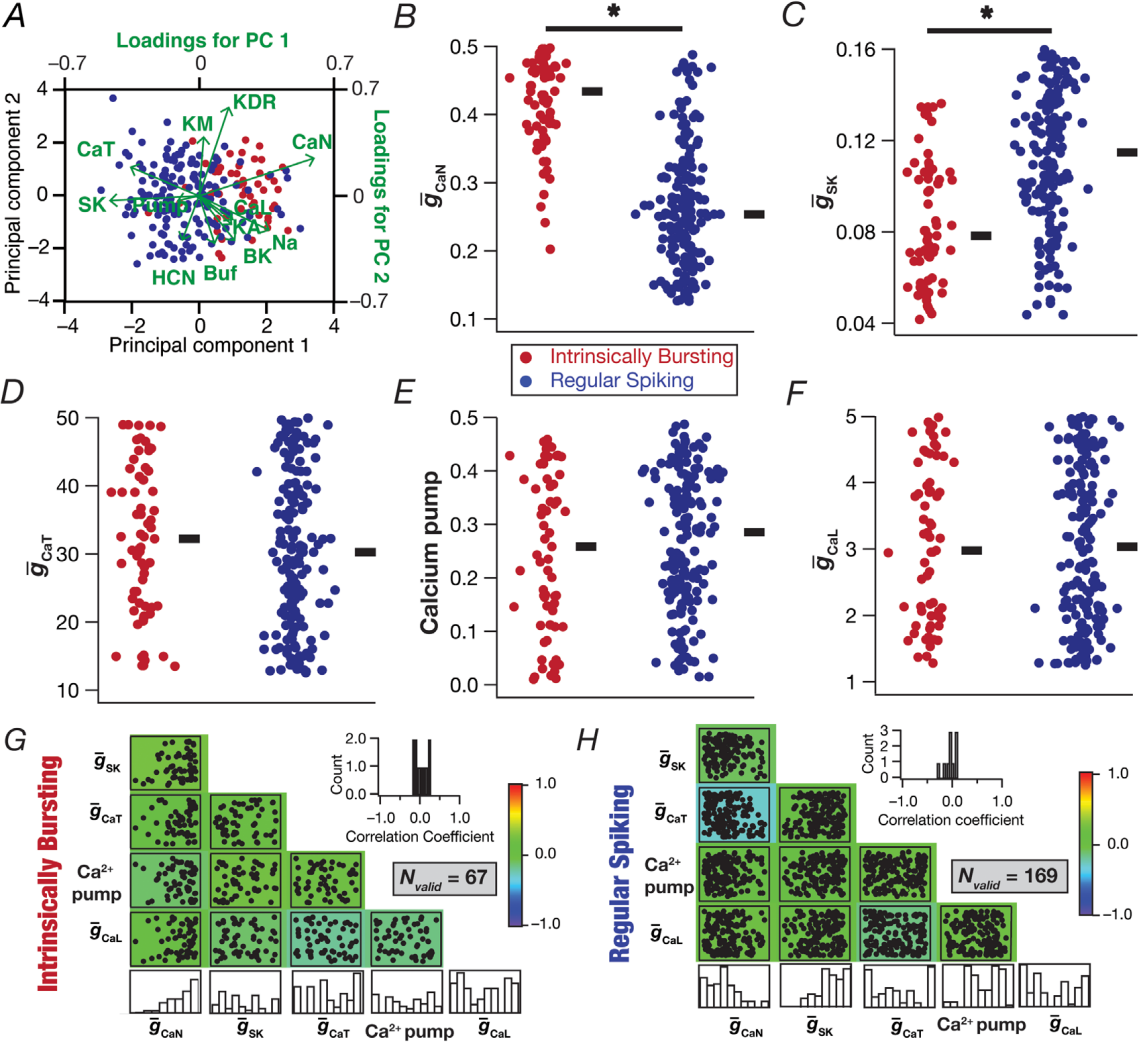
Significant differences in the expression of calcium and calcium-activated potassium channels together contribute to the distinct populations of intrinsically bursting and regular spiking models *A*, biplot showing the intrinsically bursting (*N_valid_* = 67) and regular spiking (*N_valid_* = 169) models and the projection lines indicating the relationship between the original dimension of the individual variables and their contribution to the relevant principal components. *B*–*F*, beeswarm plot showing the distribution of maximal conductance of the N-type calcium channel (CaN; *B*), maximal conductance of the calcium-activated potassium channel (SK; *C*), maximal conductance of the T-type calcium channel (CaT; *D*), the calcium pump (*E*) and the L-type calcium channel (CaL; *F*) for the intrinsically bursting and the regular spiking populations. Thick lines on the right depict respective median values. *P < 0.05, Wilcoxon signed rank test. *G* and *H*, scatterplot matrix depicting pairwise relationships between the five parameters of the 67 intrinsically bursting (*G*) and the 169 regular spiking (H) models. Scatter plots are overlaid on a heat map that shows the correlation coefficient for the respective pair of parameters. The histograms in the last row indicate the span of each parameter across the respective set of valid models. The insets represent the histograms of the 10 unique correlation coefficients plotted for the parameter pairs.

Which of the different active parameters contributed to the emergence of two distinct subpopulations of neurons? To address this, we plotted the five parameters whose loadings were along the direction of the first principal component (Fig. 11A) for all the IB and RS neurons (Fig. 11B–F). Although there were heterogeneities in these parameters across different models, emphasizing the manifestation of degeneracy in the emergence of these two subclasses, we found significant differences in CaN channel density (Fig. 11B) and the density of SK channels (Fig. 11C) between IB and RS classes of neurons. We observed widespread parametric variability and weak pairwise correlations between these five parameters in both classes of neurons (Fig. 11G and H). In addition, although the density of CaN channels was significantly higher (Fig. 11B) and that of SK channels significantly lower (Fig. 11C) in IB neurons, we did not find strong negative correlations between these two parameters in IB (Fig. 11G) or RS (Fig. 11H) neurons. These observations provided further lines of evidence for the crucial role of interactions across channel parameters and the manifestation of degeneracy in the emergence of the IB or RS class of neurons.

### Heterogeneities in CSB of RS and IB CA3 pyramidal neurons

**Figure 12:**
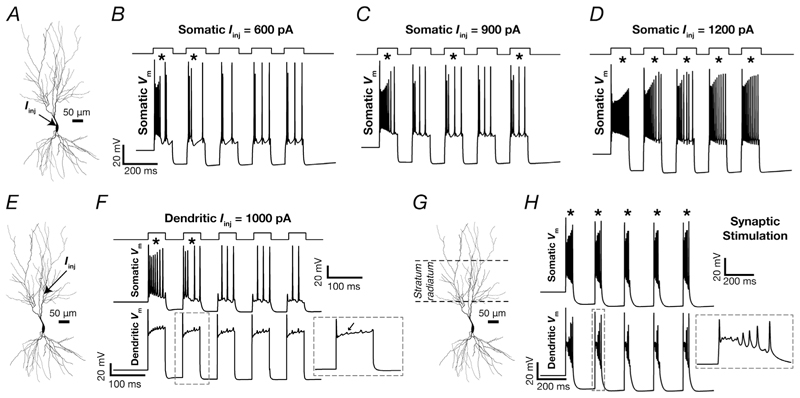
Illustrative examples showing the five different protocols used to assess complex spike bursting in CA3 pyramidal neuron models *A*–*D*, a series of five current pulses, each of 100 ms duration with an interpulse interval of 80 ms and amplitude *I_inj_*, was injected into the soma. Depicted are the resultant somatic voltage traces for *I_inj_* = 600 pA (*B*), 900 pA (*C*) and 1200 pA (*D*). *E* and *F*, a series of five current pulses, each of 50 ms duration, with an interpulse interval of 50 ms and amplitude *I_inj_* (1000 pA) was injected into a dendrite located 150 µm away from the soma (*E*). Depicted are the resultant somatic (*F*, top panel) and dendritic (F, bottom panel, at the location of current injection) voltage traces. The inset in *F* shows an enlarged view of the dendritic voltage response for the second pulse where a complex spike burst occurred. *H*, 100 randomly placed synapses within the stratum radiatum (marked in *G*) were stimulated at 5 Hz. Depicted are the resultant somatic (*H*, top panel) and dendritic (*H*, bottom panel) voltage traces. The inset in H shows an enlarged view of the dendritic voltage response for the second pulse where a complex spike burst occurred. Asterisks indicate the detection of a valid complex spike bursting event satisfying the complex spike burst validity criteria (Table 3).

An important electrophysiological signature of CA3 pyramidal neurons is the manifestation of CSB with various types of inputs arriving along the somatodendritic arbor (Hablitz & Johnston, 1981; Kowalski et al., 2016; Lazarewicz et al., 2002; Mizuseki et al., 2012; Oliva et al., 2016; Raus Balind et al., 2019; Traub et al., 1994a; Wong & Prince, 1978; Wong et al., 1979). We used the heterogeneous population of 236 model neurons to explore the roles of different ion channels and receptors in the generation of CSB in the CA3b pyramidal neurons. We used five different protocols to elicit CSB (Raus Balind et al., 2019): five somatic (Fig. 12A) current pulses of 600 (Fig. 12B), 900 (Fig. 12C) or 1200 pA (Fig. 12D); five dendritic current pulses of 1000 pA (Fig. 12E and F); and stimulation of the 100 excitatory synapses with colocalized AMPAR–NMDAR in the stratum radiatum through 5 Hz presynaptic spike trains (Fig. 12G and H). We identified the presence of CSB by placing quantitative constraints on the number of APs during the period of current injection or stimulation, the difference between the amplitudes of the first and second APs, and the amplitude of the ramp that was generated during the stimulus duration of each pulse (Table 3). Given that the first pulse (of the five pulses used for each protocol) invariably elicited valid CSB properties (e.g. Fig. 12), we excluded the first pulse from the CSB rate calculation and CSB validation process.

**Figure 13:**
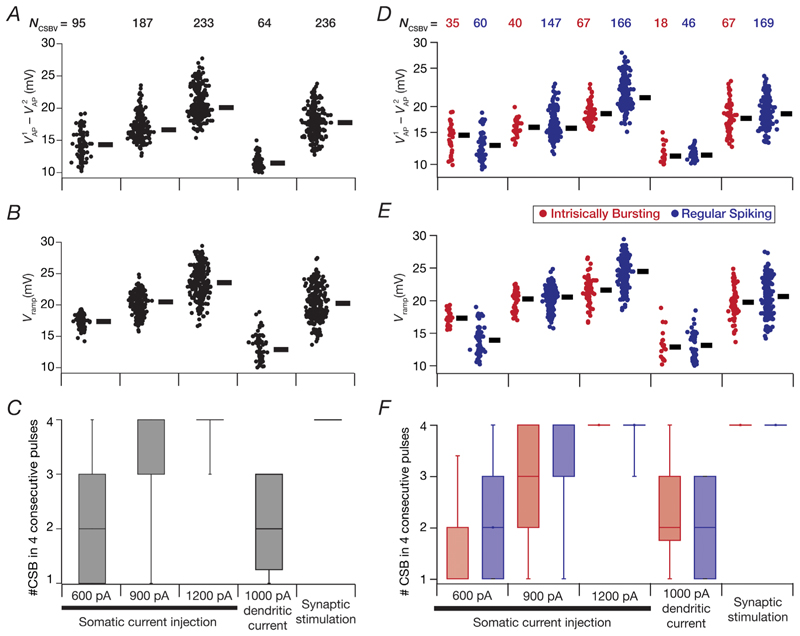
Heterogeneity in measurements of complex spike bursting across regular spiking and intrinsically bursting models with the five distinct protocols *A*–*C*, distributions of three measurements related to complex spike bursting (CSB) in all models that showed valid CSB: the average difference between the amplitude of the first ($V_{\text{AP}}^{1}$) and the second ($V_{\text{AP}}^{2}$) action potentials within a CSB (*A*), average ramp amplitude, *V_ramp_* (*B*), and the number of CSBs (*C*) observed within the five-pulse protocol (omitting the first of the five pulses). The number of models showing valid CSB (*N_CSBV_*) is provided at the top of *A* and holds for *A*–*C*. *D*–*F*, same as panels *A*–*C*, with regular spiking and intrinsically bursting models shown separately.

We repeated the same set of protocols on all 236 valid CA3 pyramidal neuron models and validated models for their ability to manifest CSB using our CSB validation criteria (Table 3). We found pronounced heterogeneity across models in their ability to manifest CSB and in the measurements that were used in their identification (Fig. 13A and B). The rate of CSB also showed considerable heterogeneity across models, in a manner that was also dependent on the specific protocol used (Fig. 13C). Overall, CSB propensity increased with increasing somatic current injection (Fig. 13C). Although the somatic current injection and the synaptic stimulation protocols were efficacious in generating CSB, the dendritic current injection protocol did not produce as many complex spike bursts owing to lower ramp voltages and smaller differences between the first and the second APs (Fig. 13A–C). In addition, even within the individual subpopulations of IB and RS models, the CSB measurements manifested considerable and comparable heterogeneities (Fig. 13D–F). There were larger proportions of IB (of the 67 valid models) and RS (of the 169 valid models) classes of neurons that showed valid CSB with increasing somatic current intensity, typically also with a larger CSB rate (Fig. 13D–F). Although the 1000 pA dendritic current pulse was not as effective as the somatic current pulses in eliciting CSB, the synaptic stimulation protocol was extremely effective in producing highly reliable CSB (Fig. 13F).

### Virtual knockout models unveiled synergistic interactions between different channels and receptors in triggering CSB in CA3 pyramidal neurons

**Figure 14:**
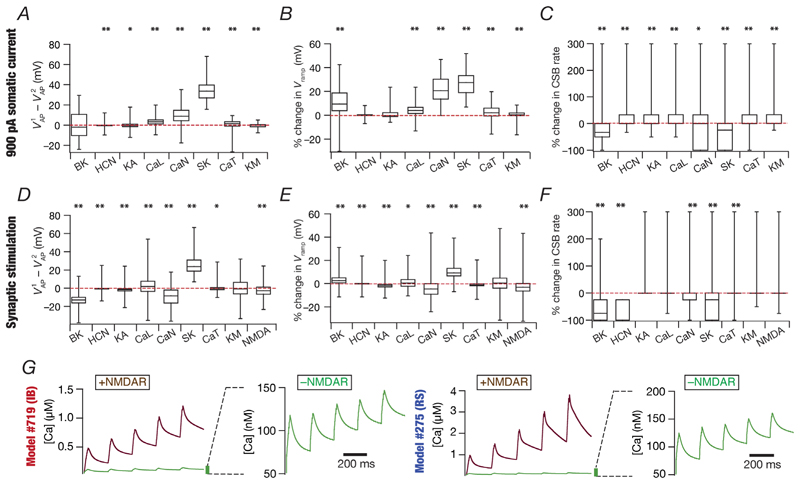
Virtual knockout models unveiled the dominance of calcium receptors, calcium-activated potassium receptors and *N*-methyl-D-aspartate receptors in the emergence of complex spike bursting with different protocols *A*–*C*, impact of virtual knockout of different ion channels on all the 187 complex spike bursting (CSB) valid models obtained with 900 pA somatic current injection. Depicted are the percentage change induced by the knockout in the average difference between the amplitude of the first ($V_{\text{AP}}^{1}$) and the second ($V_{\text{AP}}^{2}$) action potentials within a complex spike burst (*A*), average ramp amplitude (*V*_ramp_; *B*) and CSB rate (*C*). *D*–*F*, same as *A*–*C*, with CSB elicited by synaptic stimulation in 236 models that showed valid CSB. For *A*–*F*, *P < 0.05, **P < 0.01, Wilcoxon signed rank test assessed for changes with respect to a ‘no-change’ scenario. *G*, calcium traces across five pulses resulting from stimulation of the stratum radiatum synapses for two representative models that belong to the intrinsically bursting (IB) class (left) and regular spiking (RS) class (right). The impact of virtually knocking out the *N*-methyl-D-aspartate receptor (-NMDAR) on calcium is shown, providing a comparison of the calcium traces in the presence of the *N*-methyl-D-aspartate receptor (+NMDAR).

We took advantage of the heterogeneous population of CA3 pyramidal neuron models manifesting heterogeneous CSB to address the specific role of individual ion channels and receptors in eliciting CSB. To do this, we used the VKM approach (Anirudhan & Narayanan, 2015; Basak & Narayanan, 2018; Mishra & Narayanan, 2021b; Mittal & Narayanan, 2018; Mukunda & Narayanan, 2017; Rathour & Narayanan, 2014; Roy & Narayanan, 2021; Seenivasan & Narayanan, 2020) by repeating CSB protocols on each of the 236 models in the absence of individual ion channels or receptors. Specifically, we individually set the conductances of the eight active ion channels (the NaF and KDR channels were not subjected to VKM analyses to allow AP generation across all VKM models) or NMDAR permeability (Grienberger et al., 2014; Kim et al., 2012; Nunez et al., 1990; Raus Balind et al., 2019; Sipila et al., 2006; Su et al., 2001; Xu & Clancy, 2008) to zero in each valid model that showed valid CSB (Table 3) for the two protocols. We repeated the 900 pA somatic current injection (N_CSBV_ = 187 from Fig. 13A, implying 187 × 8 = 1496 VKMs for eight channels) and the 5 Hz synaptic stimulation [N_CSBV_ = 236 from Fig. 13A, implying 236 × (8 + 1) = 2124 VKMs for eight channels and NMDAR] protocols for eliciting CSB in these models. We compared CSB measurements in these VKM models with their respective base models (in which all ion channels and receptors were intact) and plotted the percentage changes across all VKMs for each ion channel/receptor (Fig. 14A–F).

We found pronounced heterogeneity in how the deletion of individual channels altered CSB properties (Fig. 14A–F). First, eliminating individual ion channels or receptors elicited a large change in certain models, whereas in other models the elimination of the same ion channel or receptor had negligible impact (Fig. 14A–F). Second, the elimination of different ion channels had distinct impacts on the same model, with considerable heterogeneity in the impact of each ion channel. Third, the calcium (CaN) and the calcium-activated potassium (SK) channels had a dominant impact in altering CSB measurements related to both intrinsically (Fig. 14A–C) and synaptically (Fig. 14D–F) elicited CSB. Finally, removal of NMDARs reduced calcium concentrations as expected at synaptic locations during synaptically induced CSB, irrespective of whether the models were RS or IB (Fig. 14G). Together, these results further emphasized ion-channel degeneracy in the emergence of CSB in CA3 pyramidal neurons, pointing to the role of synergistic interactions between several channels and receptors in CSB emergence.

## Discussion

The principal conclusion of this study is the expression of degeneracy in the emergence of characteristic somatodendritic physiological properties of CA3 pyramidal neurons, including CSB emergence with different protocols. This degeneracy spanned passive properties, ion-channel expression profiles and calcium-handling mechanisms, and from the functional perspective it covered several sub- and suprathreshold measurements of the soma and dendrites of CA3 pyramidal neurons. These findings therefore eliminate the requirement that a single set of ion channels is essential for CSB generation. Instead, our analyses show that there are several possible combinations of intrinsic mechanisms to elicit CSB, even within the same neuronal subtype, thus providing neurons with multiple degrees of freedom for CSB generation. These conclusions were arrived at based on a heterogeneous population of CA3 pyramidal neuronal models that were morphologically and biophysically realistic and were validated against several signature electrophysiological properties. Consistent with prior electrophysiological observations, we found two classes of neurons (RS and IB), with calcium and calcium-activated potassium channels playing crucial roles in mediating distinctive firing patterns. We used this heterogeneous population of valid CA3 pyramidal neurons to assess CSB using five distinct protocols and assessed the role of different ion channels and receptors in regulating CSB. Although there was a dominance of calcium and calcium-activated potassium channels in the emergence of CSB, individual deletion of none of the several channels or receptor resulted in the complete elimination of CSB across all models. Together, these analyses provided lines of evidence for the expression of degeneracy in the emergence of CSB, a signature electrophysical characteristic of CA3 pyramidal neurons with several functional roles.

### Mechanisms behind the generation of CSB

A plethora of mechanisms have been implicated in the generation of CSB across different neurons (Ashhad & Narayanan, 2016, 2019; Bastian & Nguyenkim, 2001; Condamine et al., 2018; Cueni et al., 2008; Hablitz & Johnston, 1981; Hemond et al., 2008; Izhikevich, 2007; Kadala et al., 2015; Krahe & Gabbiani, 2004; Larkum et al., 1999; Lazarewicz et al., 2002; Metzen et al., 2016; Migliore et al., 1995; Morquette et al., 2015; Narayanan & Chattarji, 2010; Perez-Reyes, 2003; Sipila et al., 2006; Stuart & Hausser, 2001; Su et al., 2001; Swensen & Bean, 2003, 2005; Tazerart et al., 2008; van Elburg & van Ooyen, 2010; Vervaeke et al., 2006; Wang et al., 2000; Williams & Stuart, 1999; Wong & Prince, 1978; Xu & Clancy, 2008; Xu et al., 2012; Yue & Yaari, 2004). Given the widespread expression of these different ion channels, it is important to recognize that these disparate mechanisms need not be mutually exclusive across different neuronal subtypes. Ion channels with overlapping activation profiles and kinetics can perform the same function of generating these complex bursts. It is important to view neural function from the perspective of a complex system, in which there are different functionally segregated subsystems (say, ion channels and receptors) that are expressed. These functionally segregated subsystems then integrate functionally to elicit a specific function (say, CSB). A crucial attribute of such a complex system is the ability of multiple combinations of such functionally segregated components to elicit the same function or produce the same output, a phenomenon that has been referred to as degeneracy. Our analyses point to the expression of ion-channel degeneracy in the emergence of CSB in hippocampal neurons, with different ion channels and receptors being capable of coming together to contribute to its emergence.

These observations, along with gradients in intrinsic properties along the different anatomical axes, imply that one-to-one mapping between individual ion channels or receptors and complex phenomena, such as the generation of CSB, should be avoided. Although it is possible that a specific ion channel has a dominant role in the emergence of CSB in a subset of CA3 pyramidal neurons, a generalization spanning all neurons, even within the same anatomical coordinates, should be avoided. For instance, our focus here was predominantly on the CA3b neurons, in which the density of HCN channels and, consequently, the manifestation of sag is relatively weak compared with CA3c pyramidal neurons (Raus Balind et al., 2019). Therefore, if the validation process involved a large sag and matched with the CA3c pyramidal neurons, the conclusions regarding the impact of HCN channels on CSB and other intrinsic properties would be different from our conclusions here.

In addition to differences in intrinsic properties, there could be important differences in excitatory and inhibitory inputs and their strengths across the different anatomical axes. It is therefore possible that the propensity for CSB could be similar across different neurons because of the balance between the excitatory inputs, the inhibitory inputs and the intrinsic excitability (IE) of the neurons [referred to as E–I–IE balance (Seenivasan & Narayanan, 2020)]. Thus, it is extremely important to assess the role of CSB in any given CA3 pyramidal neuron from a holistic perspective that accounts for the morphology of the neuron, the specific intrinsic composition of the neuron (ion channels, pumps, buffer, etc.), the synaptic inputs (excitatory and inhibitory) and their spatial and temporal activation profiles in ethological conditions, and behavioural state dependence involving neuromodulation. The behavioural state dependence is an important attribute because a state-dependent phenomena, such as neuromodulation, can alter the intrinsic properties and the synaptic inputs onto a neuron, thereby providing neurons with context-dependent flexibility of altering their ability to produce CSB (Alonso & Marder, 2019; Goldman et al., 2001; Humphries et al., 2022; Prince et al., 2016; Tropp Sneider et al., 2006; Wagatsuma et al., 2018).

From a general standpoint, the emergence of CSB is mediated by interactions between: (1) spike-generating conductances (typically, NaF and KDR); (2) a reverberatory positive feedback loop that results in a ramp-like depolarization (mediated by reverberatory conductances, such as calcium channels, persistent sodium and NMDARs); and (3) a slower negative feedback loop, which ensures that the burst is temporally limited to a few APs by introducing a hyperpolarization (mediated by slow restorative conductances, such as SK, KM and HCN). Repetitive bursts could recruit conductances that mediate post-inhibitory rebound (such as HCN). In addition to these mediating mechanisms, there are additional modulating mechanisms that regulate CSB generation. Such modulating mechanisms could be those governing neural excitability (which regulates CSB by altering the ability of the voltage ramp to elicit APs), sources of inhibition that disrupt the voltage ramp or its ability to elicit spikes (such as inhibitory synaptic inputs), and neuromodulation that alters synaptic and/or intrinsic properties of the neuron. Given the several possible combinations of mediating and modulating mechanisms, an identical set of mediating mechanisms could result in CSB in one neuron but not in another depending on the specific modulatory mechanisms expressed in these neurons. In addition, given that a neuron could express several of these mediating and modulating mechanisms, the ability to manifest similar CSB could arise from disparate combinations of several mechanisms, which could vary in a neuron-to-neuron and a context-dependent manner. Thus, it is crucial that the global structure of the parametric space spanning all factors that mediate and modulate CSB is considered while accounting for both heterogeneities and degeneracy (Alonso & Marder, 2019; Goaillard & Marder, 2021; Goldman et al., 2001; Rathour & Narayanan, 2019). Thus, the generation and analyses of a population of heterogeneous models manifesting degeneracy coupled with experimental recordings that assess CSB in different physiological conditions are essential in studying the emergence of CSB in an unbiased manner.

### Physiological and pathological implications of degeneracy in the emergence of complex spike bursting

Traditionally, the physiological relevance of bursts has been studied with reference to reliable information transmission, selectivity in transmitted information and synaptic plasticity (Lisman, 1997; Izhikevich et al., 2003; Krahe & Gabbiani, 2004; Metzen et al., 2016; Sakmann, 2017). The roles of CSB and associated dendritic plateau potentials have received renewed attention with recent demonstrations that have implicated them in behavioural time-scale synaptic plasticity (Bittner et al., 2015, 2017; Magee & Grienberger, 2020; Zhao et al., 2020), perception (Larkum, 2013; Manita et al., 2015; Takahashi et al., 2016, 2020), anaesthesia (Aru et al., 2020; Redinbaugh et al., 2020; Suzuki & Larkum, 2020), active sensing (Lavzin et al., 2012; Ranganathan et al., 2018; Xu et al., 2012) and learning (Doron et al., 2020; Larkum et al., 2022). In addition to these, the significance of bursts in pathological conditions is well established (Beck & Yaari, 2008; Kole et al., 2007; McCormick & Contreras, 2001; Su et al., 2002; Traub et al., 1994b, 1996). In this context, our demonstration of degeneracy in the emergence of CSB cautions against a search for a single biophysical mechanism that governs CSB and affects physiological outcomes or rescues pathological conditions, even within neurons of the same subtype. In executing such a search, it is crucial to account for the heterogeneous expression profiles of different mediating and modulating mechanisms, in addition to the ability of disparate combinations of these parameters to elicit similar CSB. The ability of many mechanisms to alter CSB emergence or propensity implies that perturbation of any single mechanism would result in a heterogeneous impact on CSB generation or propensity (Alonso & Marder, 2019; Mishra & Narayanan, 2021b). Thus, in assessing the impact of complex spike bursts, their blockade or generation through perturbation of any specific mechanism, it is important to record the heterogeneity across neurons and account for this in the overall analyses. Degeneracy observed in the generation of CSB, and therefore in its suppression, could also be used effectively for identifying specific drug targets that would be effective in CSB suppression but have minimal secondary and off-target effects.

### Limitations and future directions

In our analyses, we focused on CA3b pyramidal neurons, constraining models based on recordings from these neurons. However, there are lines of evidence for gradients in intrinsic properties along all anatomical axes: dorsal–ventral, proximal–distal and deep–superficial (Bilkey & Schwartzkroin, 1990; Cembrowski & Spruston, 2019; Kowalski et al., 2016; Masukawa et al., 1982; Oliva et al., 2016; Raus Balind et al., 2019; Sun et al., 2017b, 2020; Turner et al., 1995). These differences imply that pyramidal neuron CSB in different parts of the CA3 region could be mediated by disparate sets of intrinsic mechanisms, which need careful experimentation and associated populations of heterogeneous computational models across these axes. These analyses should not only account for gradients in biophysical properties, but should also account for heterogeneities in morphology (Bilkey & Schwartzkroin, 1990; Ding et al., 2020; Hunt et al., 2018; Mago et al., 2021; Narayanan & Chattarji, 2010; Raus Balind et al., 2019; Sun et al., 2017a, 2020) and afferent synaptic inputs. In addition to these, characterization of ion-channel densities and gating kinetics along the somatodendritic axis using cell-attached recordings has been minimal in CA3 pyramidal neurons. It is well established that channel densities and gating kinetics manifest gradients across the somatodendritic axis of several neuronal subtypes, providing the biophysical basis for their signature electrophysiological properties (Johnston & Narayanan, 2008; Johnston et al., 1996; Narayanan & Johnston, 2012; Nusser, 2009, 2012). Therefore, it is essential that model populations are refined to incorporate detailed electrophysiological characterization of the different ion-channel subtypes in CA3 pyramidal neuron dendrites along each of the anatomical axes mentioned above. Such analyses could also explore heterogeneities in the ionic basis and potential ion-channel degeneracy in an identified subclass of CA3 pyramidal neurons that manifest spike frequency adaptation (Hemond et al., 2008).The search for populations of models that show spike frequency adaptation and heterogeneities therein should incorporate measurements of adaptation that are derived from the temporal evolution profiles of spike patterns with different amplitudes of current injection (Hemond et al., 2008).

Of the disparate mechanisms that have been implicated in the emergence of CSB, our study focused on intrinsic properties and synaptic activation. However, it is important that the roles of different neural circuit mechanisms and heterogeneities therein are assessed to gain an understanding of the emergence of CSB *in vivo*. Prominent among these are pathway interactions between different sets of inputs, the impact of timed inhibitory inputs and E–I balance, in addition to the astrocytic origins of plateau potentials and bursts. These analyses are best performed in a heterogeneous network of morphologically realistic CA3 pyramidal neuron models, also endowed with interacting populations of heterogeneous interneurons and astrocytes, receiving afferent inputs from the dentate gyrus and the entorhinal cortex. In parallel, detailed electrophysiological experiments should be conducted regarding each of these different neural-circuit mechanisms involved in the emergence of CSB. For instance, although it is known that astrocytic activation results in bursts in other neuronal subtypes, it is important to test this in CA3 pyramidal neurons (Ashhad & Narayanan, 2016, 2019; Condamine et al., 2018; Kadala et al., 2015; Morquette et al., 2015). These heterogeneous network models could then be used to explore state and context dependence using the neuromodulatory influence on neural circuit properties as the link. These disparate routes and additional mechanisms in such a model would then provide an ideal substrate for exploring the manifestation of degeneracy, involving all neural circuit components, in the emergence of CSB. Such heterogeneous network models that are validated explicitly against electrophysiological experiments would also provide a substrate for assessing the role of active dendritic properties on local field potentials, including the signature sharp wave ripples that are dependent on population bursts in the CA3 region (Buzsaki, 2015; Sinha & Narayanan, 2022; Sullivan et al., 2011). Cellular subtypes (neurons and astrocytes) and their intrinsic properties, network connectivity and synaptic properties are typically very different across different brain regions. Therefore, generalization of the conclusions drawn here to CSB in other brain regions should be done only after careful exploration of neural-circuit properties and associated heterogeneities in the brain region of interest.

Future analyses could investigate the role of CA3 CSB in learning, plasticity and behaviour using *in vitro* and *in vivo* electrophysiological experiments (Aru et al., 2020; Bittner et al., 2015, 2017; Doron et al., 2020; Larkum, 2013; Larkum et al., 2022; Lavzin et al., 2012; Magee & Grienberger, 2020; Manita et al., 2015; Ranganathan et al., 2018; Redinbaugh et al., 2020; Suzuki & Larkum, 2020; Takahashi et al., 2016, 2020; Xu et al., 2012; Zhao et al., 2020) and computational analyses involving biophysically constrained plasticity rules dependent on CSB (Milstein et al., 2021). Given that CA3 pyramidal neurons receive a multitude of inputs from different brain regions (entorhinal cortex, dentate gyrus and CA3 region), it would be interesting to assess the emergence of CSB through pathway interactions and heterogeneities therein. These experiments, apart from providing further evidence for the importance of CSB in ethological conditions, could also explore routes to harness the manifestation of degeneracy as a tool for designing experiments. Such analyses could explore the role of heterogeneous synaptic distributions, different morphological characteristics and ion-channel distributions on place-cell tuning, dendritic spike generation and information transmission in CA3 pyramidal neurons through rate and phase codes (Basak & Narayanan, 2018, 2020; Roy & Narayanan, 2021; Seenivasan & Narayanan, 2020). Finally, these experiments could probe the several disparate routes that could regulate CSB generation and propensity, with the aim of altering physiological outcomes or reversing pathological conditions.

## Figures and Tables

**Table 1:** Model parameters, with their base values and the range spanned for the unbiased stochastic search

	Parameter (unit)	Symbol	Base value	Range
Passive properties (uniform across the neuron)				
1	Membrane resistance (kΩm^−2^)	*R_m_*	60	60–100
2	Axial resistance (Ω cm)	*R_a_*	200	150–400
Active properties				
Spike-generating channels (uniformly distributed)				
3	Maximal conductance for NaF (mS cm^−2^)	${\bar{g}}_{\text{Na}}$	15	7.5–30.0
4	Maximal conductance for KDR (mS cm^−2^)	${\bar{g}}_{\text{KDR}}$	11	5.5–22.0
HCN channel (increase from soma to distal apical dendrite)				
5	Maximal conductance (μS cm^−2^)	${\bar{g}}_{\text{h}}$	1	0.5–6.0
A-type potassium channel (linear increase with distance along apical dendrite)				
6	Maximal conductance (mS cm^−2^)	${\bar{g}}_{\text{KA}}$	0.1	0.05–0.20
L-type calcium channel (perisomatic distribution)				
7	Maximal conductance (mS cm^−2^)	${\bar{g}}_{\text{CaL}}$	2.5	1.25–5.00
T-type calcium channel (uniformly distributed)				
8	Maximal conductance (mS cm^−2^)	${\bar{g}}_{\text{CaT}}$	0.25	0.125–0.500
N-type calcium channel (uniformly distributed)				
9	Maximal conductance (mS cm^−2^)	${\bar{g}}_{\text{CaN}}$	2.5	1.25–5.00
M-type potassium channel (perisomatic distribution)				
10	Maximal conductance (mS cm^−2^)	${\bar{g}}_{\text{KM}}$	0.01	0.005–0.02
Large conductance calcium-activated potassium BK channel (uniformly distributed)				
11	Maximal conductance (mS cm^−2^)	${\bar{g}}_{\text{BK}}$	0.8	0.4–1.6
Small conductance calcium-activated potassium SK channel (uniformly distributed)				
12	Maximal conductance (mS cm^−2^)	${\bar{g}}_{\text{SK}}$	0.5	0.25–1.00
Calcium-handling mechanisms				
13	Total calcium pump	totpp	0.2	0.01–0.50
14	Total calcium buffer	totbuf	1.2	0.1–2.50

Parametric search ranges were chosen to be around the base model value, with most ranges chosen to be 0.5-2 times that of the respective base model value. Some parameters were set to span a different range based on physiological constraints or the need to expand the ranges (e.g. HCN conductance).

**Table 2:** Intrinsic measurements of CA3 pyramidal neurons and their respective electrophysiological bounds

	Measurement	Symbol	Lower bound	Upper bound
1	Resting membrane potential (mV)	*V*_RMP_	−70	−60
2	Standard deviation (μV)	*V*_SD_	0	0.01
3	Input resistance (MΩ)	*R*_in_	60	150
4	Sag ratio	*Sag*	0.94	1
5	Resonant frequency (Hz)	*f*_R_	1	1.76
6	Strength of resonance	*Q*_R_	1	1.1
7	Impedance amplitude (MΩ)	|*Z*|_max_	65	150
8	Firing rate at 250 pA (Hz)	*f*_250_	4	10
9	AP amplitude at soma (mV)	$V_{\text{AP}}^{0}$	90	110
10	AP amplitude at 150 μm (mV)	$V_{\text{AP}}^{150}$	70	90
11	AP amplitude at 300 μm (mV)	$V_{\text{AP}}^{300}$	60	80

These measurements were derived from electrophysiological recordings reported by Hemond et al. (2008), Kim et al. (2012) and Raus Balind et al. (2019) and were set to account for a large proportion (~80%) of the reported distributions of the respective measurement. The ranges were computed from the reported mean and SD values or from the plotted distributions. Abbreviation: AP, action potential.

**Table 3:** Measurements and their bounds used to validate complex spike bursting

	Measurement	Lower bound	Upper bound
1	Number of action potentials in the complex spike burst	3	–
2	$\Delta V_{\text{AP}}=V_{\text{AP}}^{1}-V_{\text{AP}}^{2}(\text{mV})$	10	30
3	Ramp amplitude, *V*_ramp_ (mV)	10	30

## Data Availability

This is a computational study, hence no experimental data were generated as part of this study. All computational outcomes and analyses required for assessment of thismanuscript are available as part of the figures and tables.

## Key points

- An unbiased stochastic search algorithm yielded a heterogeneous population of morphologically and biophysically realistic CA3 pyramidal neuronal models matching several signature electrophysiological characteristics.
- Two functional subclasses of valid models were identified with intrinsically bursting (IB) and regular spiking (RS) characteristics, which exhibited differential localization within the parametric space with linear and non-linear dimension reduction analyses.
- Calciumand calcium-activated potassium channels distinguished IB fromRSmodels, apart from playing dominant roles in the emergence of complex spike bursting (CSB).
- The impact of deleting individual ion channels or N-methyl-d-aspartate receptors was variable across different models and differential for each channel/receptor, pointing to ion-channel degeneracy in the emergence of CSB.
- Biological heterogeneities across different neurons of the same subtype, ion-channel degeneracy and state-dependent changes (involving activity-dependent plasticity, pathology, and neuromodulation of intrinsic and synaptic properties) need to be considered carefully in assessing the propensity and dynamics of CSB in different neuronal subtypes.
